# Inequities in Organ Donation and Transplantation Among Immigrant Populations in Italy: A Narrative Review of Evidence, Gaps in Research and Potential Areas for Intervention

**DOI:** 10.3389/ti.2023.11216

**Published:** 2023-08-11

**Authors:** Alessandra Agnese Grossi, Francesca Puoti, Lucia Masiero, Alessia Troni, Tiziana Cianchi, Umberto Maggiore, Massimo Cardillo

**Affiliations:** ^1^ Center for Clinical Ethics, Department of Biotechnologies and Life Sciences, University of Insubria, Varese, Italy; ^2^ Department of Human Sciences, Innovation and Territory, University of Insubria, Como, Italy; ^3^ Italian National Transplant Center (CNT), Istituto Superiore di Sanità, Rome, Italy; ^4^ Nephrology Unit, Dipartimento di Medicina e Chirurgia, Università di Parma, Parma, Italy

**Keywords:** organ donation, organ transplantation, migrants, ethnic minorities, inequities

## Abstract

Immigrants from outside Europe have increased over the past two decades, especially in Southern European countries including Italy. This influx coincided with an increased number of immigrants with end-stage organ diseases. In this narrative review, we reviewed evidence of the gaps between native-born and immigrant populations in the Organ Donation and Transplantation (ODT) process in Italy. Consistent with prior studies, despite the availability of a publicly funded health system with universal healthcare coverage, non-European-born individuals living in Italy are less likely to receive living donor kidney transplantation and more likely to have inferior long-term kidney graft function compared with EU-born and Eastern European-born individuals. While these patients are increasingly represented among transplant recipients (especially kidney and liver transplants), refusal rates for organ donation are higher in some ethnic groups compared with native-born and other foreign-born referents, with the potential downstream effects of prolonged waiting times and inferior transplant outcomes. In the process, we identified gaps in relevant research and biases in existing studies. Given the Italian National Transplant Center’s (CNT) commitment to fighting inequities in ODT, we illustrated actions taken by CNT to tackle inequities in ODT among immigrant communities in Italy.

## Introduction

“Diversity, equity and inclusion” are emergent issues in transplantation healthcare. Studies, including a manifesto of the European Society for Organ Transplantation (ESOT), have emphasized the ethical duty to reduce inequities among the most vulnerable and/or socially deprived communities, including immigrant groups [1–4]. Europe ranks first globally as host for international immigrants (82 million) [5]. In Europe, most individuals with an immigration background are regular residents who benefit from consolidated healthcare coverage. Although evidence remains limited in Europe, inequities have been described in end stage kidney disease (ESKD) treatment [6], waiting list (WL) accessibility [7–9], likelihood of preemptive and living donor kidney transplant (LDKT) [10–14] and outcomes (i.e., graft survival and function) of kidney transplant (KT) among immigrant populations relative to their European-born referents [6, 15, 16]. Studies have also noted that these populations are increasingly represented among patients requiring transplantation. At the same time, these groups have higher rates of refusal for deceased organ donation when compared to native-born and other foreign-born counterparts, with the potential for detrimental effects on waiting times and outcomes of transplantation [17–19].

Immigrants are a heterogeneous group. Features may vary among individuals as a result of the reasons for and circumstances of migration, migration pathways, and across countries depending on migration history, immigration policies, social security systems, and other societal features [20]. However, these populations are often exposed to structural inequities. Structural inequities may increase health risks at each stage of the migration process, namely, before migration takes place, during the migration process, during integration, and after return to the home country. Risks may increase because of cultural, linguistic and relational difficulties, diminished socioeconomic conditions, discrimination, inferior knowledge of healthcare systems and administrative regulations in host countries, and the lack of culturally competent healthcare services [3, 21–25]. The interplay among these factors may lead to inequities in healthcare accessibility and quality, even in countries with universal healthcare coverage [26, 27].

Italy ranks third in Europe in the total number of regular residents with non-EU citizenship (5.2 million) [28]. Of these, the most numerous are the Romanian, Albanian, Moroccan, and Chinese communities (see resident immigrant groups based on citizenship in Supplementary Table S1). Given the recent surge in immigration, adult immigrant and ethnic minority groups in Italy overlap [29].

In Italy, immigrant populations are generally identified as vulnerable groups. For instance, non-European-born individuals in Italy are more likely to be socioeconomically disadvantaged, with difficulties in oral and written communication, and, relative to individuals who have migrated to other EU countries, with a lower educational level [26, 30]. Most immigrants are younger and healthier when compared to native-born referents. Similar to European data [31–33], a large segment of first-generation immigrants in high migratory pressure areas in Italy has ESKD (7.6%–35%) [34–36] and liver disease (9.2%) [37]. Studies reveal that these populations present significantly higher rates of diabetes, obesity (especially among subjects of South-east Asian and Northern African origin) and other risk factors (i.e., hypertension) for the progression toward organ failure (especially ESKD) requiring replacement therapies [27, 38, 39]. Italy has a universal healthcare system which guarantees organ transplantation regardless of ethnicity, immigration background, religious beliefs, gender, socio-economic status, health literacy, linguistic difficulties, or cultural diversity. However, evidence from other healthcare settings has shown inequities in accessibility, quality and outcomes of care among immigrant populations [26, 27]. Equity is a major principle in organ transplantation; ensuring equity in ODT requires to first examine whether inequities are present. Tackling inequities is critical to ensure that all people achieve the best possible health outcomes, and addressing inequities is key for the trustworthiness and transparency of transplant systems [40].

The objective of this narrative review is to determine whether gaps exist between immigrants and native-born populations in ODT in Italy. In the process, we seek to identify gaps in research and potential areas for intervention, and illustrate actions that CNT has undertaken thus far to tackle inequities in ODT among immigrant communities in Italy.

## Methods

### Search Strategy

We searched PubMed for case-control studies, retrospective and prospective cohort studies, and cross-sectional studies addressing inequities in ODT in immigrant populations in Italy. We searched for articles written in English and Italian published between 1st January 2000 and 28th March 2023 using the following search string: (immigrants OR migrants OR immigration) AND (“organ don*” OR “organ transplant*” OR transplant*) AND (Italy OR Italian). The references of the selected articles were scrutinized for additional references.

### Definitions

For the purposes of this narrative review, we adopt the following definitions of key terms:

Health inequities: “potentially avoidable differences in health, or in health risks that policy can influence, between groups of people who are more or less advantaged socially, *which* … systematically place socially disadvantaged groups at further disadvantage on health” [41]. This definition, grounded in ethical and human rights principles, emphasizes the subcategory of health differences indicative of social injustice, which distinguishes health inequities from other health differences.

Migrant: “any person who is outside a State of which he or she is a citizen or national, or, in the case of a stateless person, his or her State of birth or habitual residence. The term includes migrants who intend to move permanently or temporarily, and those who move in a regular or documented manner as well as migrants in irregular situations” [42]. According to the European Commission, the migrant category excludes persons who travel for tourism or business purposes and excludes intra-EU mobility [43].

Ethnicity: “the social group a person belongs to, and either identifies with or is identified with by others, as a result of a mix of cultural and other factors including language, diet, religion, ancestry, and physical features” that are shared by individuals in the same group [44].

As recommended by prior reports [45], both the features related to “ethnicity” (including country of origin or descent) and “immigrant status” (or family history of immigration) should be considered for the purposes of studies in relation to ODT [45]. For instance, the European Public Health Association contends that, although immigration includes also elements of ethnicity, “visible minorities” are likely to experience more significant inequities relative to their White referents, similar to immigrants [46].

### Eligibility Criteria

We included articles meeting the following criteria: addressing organ donation and/or transplantation and including individuals with an immigration background residing in Italy. We excluded articles addressing non-organ transplantation (i.e., tissue, blood or cell donation), systematic reviews, literature reviews, and case reports.

### Data Extraction

Following identification of eligible articles, the following data were extracted and included in a descriptive table presenting: authors and publication year, age group, Italian area where studies were performed, study design and analysis, main estimates, and comment to study findings.

## Results

The PubMed search yielded 66 articles. Of these, 59 were excluded after screening of title and abstract as not matching the inclusion criteria. Following full text review, the remaining 7 articles were assessed for eligibility and were included in the review. One article was included from the reference section of a selected article.

### Study Characteristics

The descriptive details and main findings of the eight included articles are summarized in Table 1. The period of data collection varied between 1994–2001 [48] and 2010–2020 [49]. Studies were published between 2004 and 2022. There were two survey studies [35, 36] but most had a retrospective design [34, 47–51]. Of these, five were cohort studies based on national [47, 49, 50] and regional registries [34, 51], and one was a single center study [48]. Articles were categorized according to the following time-points of the ODT process, as described below:

• ESKD treatment,
• access to the transplant waitlist (WL),
• likelihood of transplant,
• outcomes of transplant,
• transplantation and refusal rates to deceased organ donation.

**TABLE 1:** Current evidence of inequities in organ donation and transplantation among immigrant and ethnic minority populations in Italy.

	Study ID	Italian area	Study design and analysis	Countries of origin/Ethnicity	Age group	Main estimates	Comment
ESKD treatment	[47]	Nationwide	Retrospective cohort study (2007–2016); on native-born (*n* = 328) and RFPs (*n* = 120). Logistic regression analysis	South America [mother (10%); father (7.5%)]; Asia [mother (15%); father (14.2%)]; North Africa [mother (25.8%); father (27.5%)]; Central Africa [mother (10%); father (10%)]. 82.5% of mothers and 80% of fathers of immigrant children came from low- or medium-income countries	Pediatric	RFPs increased from 23% to 30.3% (*p* = 0.08) [period 1 (2007–2011) vs. period 2 (2012–2016)]. RFPs were younger (6.7 vs. 9.4 years, *p* = 0.025) and less often treated with preemptive KT (3.3% vs. 13.4%, *p* = 0.009) than native-born Pts. The percentage of preemptive KT increased from period 1 to 2 in RFPs only (8.4%–18.6%, *p* = 0.006)	5-Year Pt survival [RFPs vs. native-born (87.4% vs. 89.7%, *p* = 0.35)], waiting time to KT (2.2 vs. 2.4 years as a median, *p* = 0.45), and dialysis modality survival did not differ between native-born and RFPs
	[36]	Lombardy	Survey study (2015) of Pts with ESKD (*n* = 7,463) of whom 8.41% (*n* = 628) were non-EU-born. Unadjusted descriptive analysis	Eastern Europe (14.9%), North Africa (25.5%), Sub-Saharan Africa (17%), Latin America (11.8%), Asia (30.4%), Oceania (0.5%)	Adult	Relative to EU-born Pts, non-EU-born Pts were younger (Aged 41–40 vs. 61–80), more often on HD (93.1% vs. 87.7%, *p* < 0.001) and less on PD (6.9% vs. 12.3%, *p* < 0.001), and late referral [6 months prior to dialysis (48%), 1–6 months (20.2%), over the last 30 days (30.8%)]	The distribution of ethnicity of immigrant Pts was different from the total foreign population as reported by census data. Data were not adjusted for potential confounders
	[34]	Lazio	Retrospective cohort study (2004–2012); KT eligibility and WL registration rates of immigrant (*n* = 365) and native (*n* = 4,411) Pts with ESKD. Unadjusted descriptive analysis	Romania [*n* = 39 (10.7%)], Philippines [*n* = 38 (10.4%)], Egypt [*n* = 23 (6.3%)], Libya [*n* = 16 (4.4%)], Tunisia [*n* = 15 (4.1%)], Ethiopia [14 (3.8%)], Albania [12 (3.3%)], Bangladesh [12 (3.3%)], Morocco [12 (3.3%)], France [11 (3.0%)], Serbia-Montenegro [10 (2.7%)], Nigeria [10 (2.7%)], other (<10 Pts) [153 (41.9%)]	Adult	Compared with Italians, immigrant Pts were younger (53.8 ± 16.3 vs. 68.7 ± 13.6), more frequently women (42.7% vs. 37.7%), HbsAg-positive (18.1% vs. 13.9%), not vaccinated if HBV susceptible (26.8% vs. 20.9%), late referral (34.9% vs. 18.9%, *p* < 0.001), suitable for KT (21.7% vs. 9.9%), with.higher survival probability at 1 (91.9% vs. 84.7%) and 5 years (74.6% vs. 51.5%) since dialysis start (HR = 0.71; CI 95%:0.58–0.87)	Only mortality risk was adjusted for multiple potential confounders
	[35]	Piedmont	Survey study across 19/25 dialysis facilities; on immigrant Pts (*n* = 93) with ESKD on chronic dialysis treatment. Unadjusted descriptive analysis	Morocco (*n* = 26), Albania (*n* = 15), Romania (*n* = 9), Senegal (*n* = 7), Nigeria (*n* = 5), other (<5) (*n* = 31)	Adult	At presentation, most Pts were young (mean age 46 ± 14 years), on HD (87%); late referral (38%) or starting dialysis in emergency (17%). No difference in HCV, HBV and HIV incidence relative to natives. Most Pts had low-level knowledge of Italian (56%), were regular foreign citizens (69%), temporary foreign workers (19%), or had a residence permit (9%)	Social and relational problems are more challenging than clinical aspects and call for new organizational models to manage this growing population with ESKD. Rates were not adjusted and the study did not report control group data. A national cohort study controlling for potential confounders is missing
		[48]	Lombardy	Retrospective single center study (1994–2001) on Pts (*n* = 12) from developing countries and 59 native Pts with ESKD. Unadjusted descriptive analysis	Philippines (*n* = 5), Egypt (*n* = 4), Morocco, Mauritius, Sri Lanka (*n* = 1)	Adult	Pts from developing countries on dialysis differ from the native dialysis population in younger age, causes of kidney failure, late referral, higher infection rates (67%), and clinical complications due to Pts’ visits to home countries. At follow-up (45.3 ± 32.0 months), 5 Pts continued on HD, 2 were on PD, and 4 received KT and 1 a KT and LT.	Data were not adjusted for potential confounders
	Access to the transplant waiting list	[36]	Lombardy	Survey study (2015) of Pts with ESKD (*n* = 7,463) of whom 8.41% (*n* = 628) were non-EU-born. Unadjusted descriptive analysis	Eastern Europe (14.9%), North Africa (25.5%), Sub-Saharan Africa (17%), Latin America (11.8%), Asia (30.4%), Oceania (0.5%)	Adult	WL registration (34.8% vs. 18%, *p* < 0.01) non-EU-born vs. EU-born	Data were not adjusted for potential confounders
		[34]	Lazio	Retrospective cohort study (2004–2012); of KT eligibility and WL registration rates of immigrant and native Pts with ESKD. Unadjusted descriptive analysis	Romania [*n* = 39 (10.7%)], Philippines [*n* = 38 (10.4%)], Egypt [*n* = 23 (6.3%)], Libya [*n* = 16 (4.4%)], Tunisia [*n* = 15 (4.1%)], Ethiopia [14 (3.8%)], Albania [12 (3.3%)], Bangladesh [12 (3.3%)], Morocco [12 (3.3%)], France [11 (3.0%)], Serbia-Montenegro [10 (2.7%)], Nigeria [10 (2.7%)], other (<10 Pts) [153 (41.9%)]	Adult	Unadjusted KT eligibility (31.2% vs. 29.5%, *p* = 0.57) and WL registration (93.9% vs. 91.6%, *p* = 0.43) of immigrant and native Pts	Unadjusted rates do not account for immigrants being younger and with lower prevalence of comorbidities compared to EU counterparts. A national cohort study controlling for potential confounders is not available yet.
[35]		Piedmont	Survey study across 19/25 dialysis facilities; on immigrant Pts (*n* = 93) with ESKD on chronic dialysis treatment. Unadjusted descriptive analysis	Morocco (*n* = 26), Albania (*n* = 15), Romania (*n* = 9), Senegal (*n* = 7), Nigeria (*n* = 5), other (<5) (*n* = 31)	Adult	Active status on the WL (27%); in the process of being evaluated (23%); inactive (2%); not yet considered for KT (46%)	The study did not report control group data
Likelihood of transplantation		[49]	Nationwide	Retrospective cohort study (2010–2020); on EU-born (*n* = 21,624), Eastern European-born (*n* = 606) and non-European-born (*n* = 1,944). Competing risk analysis	Asian [614 (31.6%)], Hispanic [297 (15.3%)], Sub-Saharan Africa [525 (27%)], North Africa and Middle East [508 (26.1%)]	Adult	LDKT adjusted relative probability of non-European-born vs. Eastern European-born 0.51 (95% CI: 0.33–0.79; *p* = 0.002); of non-European-born vs. EU-Born: 0.65 (95% CI: 0.47–0.82; *p* = 0.001)	Immigration status did not affect the rate of DDKT or permanent WL withdrawal
			[47]	Nationwide	Retrospective cohort study (2007–2016); on native-born (*n* = 328) and RFPs (*n* = 120). Logistic regression analysis	South America [mother (10%); father (7.5%)]; Asia [mother (15%); father (14.2%)]; North Africa [mother (25.8%); father (27.5%)]; Central Africa [mother (10%); father (10%)]. 82.5% of mothers and 80% of fathers of immigrant children came from low- or medium-income countries	Pediatric	Belonging to the RFPs group was associated with a significantly lower probability of receiving a preemptive KT [ RFPs vs. native-born (3.3% vs. 13.4%, *p* = 0.009)]	5-Year Pt survival [RFPs vs. native-born (87.4% vs. 89.7%, *p* = 0.35)], waiting time to KT (2.2 vs. 2.4 years as a median, *p* = 0.45), and dialysis modality survival did not differ between native-born and RFPs
Outcomes of transplantation	[50]	Nationwide	All adult deceased KTR in Italy (2010–2015) followed-up until death, dialysis or 5-Years post-transplantation: EU-born (*n* = 6,346), Eastern European-born (*n* = 161), and non-European-born (*n* = 490). Joint longitudinal survival analysis	Asian [142 (29.0%)], Hispanic [68 (13.9)], African [135 (27.6%)], North Africa and Middle East [144 (29.4%)]	Adult	Compared to EU-born KTRs, in non-European-born KTRs adjusted average yearly eGFR decline was −0.96 mL/min/year (95% confidence interval: −1.48 to −0.45; *p* < 0.001), whereas it was similar in Eastern European-born KTRs [+0.02 mL/min/year (−0.77 to +0.81; *p* = 0.96)]	Adjusted 5-Year transplant survival did not statistically differ between non-European-born, Eastern European-born, and EU-born. In those surviving beyond 1-Year, it was 91.8% in EU-born (95% CI: 87.1–96.8), 92.5% in Eastern European-born (86.1–99.4), and 89.3% in non-European-born KTRs (83.0–96.0)
Transplantation and non-refusal rate to deceased donation	[51]	Piedmont	Retrospective cohort study (2004–2011) of brain deaths and non-refusal rates among immigrant groups in Piedmont: 126/178L (7%) brain deaths among immigrant groups from 43 different countries. 222/2,914 (7%) Tx were performed for immigrants including liver (*n* = 66), kidney (*n* = 130), heart (*n* = 21), and lung (*n* = 5). Unadjusted descriptive analysis	Unspecified	Adult	The Romanian community was the most favourable towards donation (78.8%), vs. Moroccan (25%) and Albanian (33%) which were the least favourable	Not all individuals with an immigration background have the same non-refusal rates. In contrast, non-refusal rates are lower in some ethnic minority groups relative to others. Studies are missing regarding refusal rates at the national level in Italy by immigration background and ethnicity. Studies should determine the multiple intersecting factors underlying this phenomenon

DDKT, deceased donor kidney transplant; eGFR, estimated Glomerular Filtration Rate; ESKD, end stage kidney disease; EU, European Union; KT, kidney transplant; KTR, kidney transplant recipient; LDKT, living donor kidney transplant; LT, liver transplant; PD, peritoneal dialysis; RFP, resident foreign patient; WL, waiting list.

### Main Findings of Included Studies

#### End Stage Kidney Disease Treatment

There were five studies on the treatment of ESKD. Of these, most addressed the adult patient population [34–36, 48], except one that focused on pediatric patients [47]. With the exception of two survey studies [35, 36], the remaining articles had a retrospective design [34, 47, 48], of which two were based on a regional [34] and national registry [47]. Only three studies specified the patients’ countries of origin beyond broader ethnicity categories [34, 35, 48]. All studies reported that immigrant patients with ESKD on chronic dialysis treatment are younger relative to their native-born referents, have a regular residency permit, and most frequently originate from Northern Africa and Asia (where this was explicitly stated, patients from Northern Africa originated mostly from Egypt, Morocco, Libya, and Tunisia; patients from Asia chiefly from the Philippines and Bangladesh) [34, 35, 48]. In adults, referrals were more often delayed [34–36, 48], whereas this information was missing in the pediatric study [47]. The 1- (74.6% vs. 51.5%) and 5-year (91.9% vs. 84.7%) patient survival after dialysis start was significantly higher among immigrants in Lazio [34]; no differences were detected between the children born of immigrant parents and their native-born counterparts (87.4% vs. 89.7%, *p* = 0.35) [47]. Two studies noted a higher rate of clinical complications following visits to home communities [35, 48]. There were no national cohort studies (prospective or retrospective) controlling for potential confounders.

#### Access to the Transplant Waiting List

Studies examining the association between immigrant status and access to the transplant WL (*n* = 3) were focused on adult patients pursuing KT in the Italian North-Western (Lombardy and Piedmont) [35, 36] and Central areas (Lazio) [34]. The studies from Lombardy and Piedmont were surveys. The study from Piedmont found that, irrespective of the younger age and better clinical conditions relative to natives, a large proportion of immigrant patients with ESKD is not yet considered for KT (46%), is in the process of being evaluated (23%) or is inactive (2%). The study reported that, in many cases (40%), language barriers compromise patient-provider communication, leading to impairment of informed consent and reducing adherence to prescribed medical and dietary regimens. Pending regularization status and other socioeconomic factors including poverty and poor housing quality are reported as factors with the potential to reduce the chance for these patients to be waitlisted. Periodic visits to home countries and associated exposures to endemic infections and/or undertreatment of ESKD are commonly observed [35]. KT eligibility (31.2% vs. 29.5%, *p* = 0.57) and WL registration (93.9% vs. 91.6%, *p* = 0.43) rates are comparable between immigrants and natives in Lazio [34]; WL registration is significantly higher among patients from non-EU countries relative to EU-born referents in Lombardy (34.8% vs. 18%, *p* < 0.01) [36]. National cohort studies controlling for potential confounders are missing.

#### Likelihood of Transplant

Two retrospective national cohort studies of the adult and pediatric populations examined the association between immigration background and likelihood of KT [47, 49]. Competing risk analysis of adult patients waitlisted for KT revealed that non-European immigration background (i.e., from non-EU countries beyond Eastern Europe - excluding North America and Oceania) is associated with a diminished likelihood to receive LDKT [adjusted relative probability of non-European-born vs. Eastern European-born 0.51 (95% CI: 0.33–0.79; *p* = 0.002); of non-European-born vs. EU-Born: 0.65 (95% CI: 0.47–0.82; *p* = 0.001)]. In contrast, the study found that immigrant status does not affect the rate of deceased donor KT or permanent WL withdrawal [49]. The study of pediatric patients found that belonging to the immigrant group is associated with a significantly lower probability to receive a preemptive KT (OR 0.25, 95% CI 0.08–0.72, *p* = 0.011), whereas waiting time to KT does not differ between native-born and immigrant patients (2.2 vs. 2.4 years median, *p* = 0.45) [47]. This study was unique in highlighting that the majority of mothers (82.5%) and fathers (80%) of immigrant children originated from low- or medium-income countries.

#### Outcomes of Transplantation

A retrospective cohort study of the Italian National Transplantation Network assessed the association of immigration background with KT outcomes [50]. The study found that non-European immigration background (i.e., from non-EU countries beyond Eastern Europe—excluding North America and Oceania) is associated with worse long-term kidney graft function decline following KT relative to EU-born and Eastern-European born counterparts. Compared to EU-born KT recipients, in non-European-born KT recipients, the adjusted average yearly eGFR decline was −0.96 mL/min/year (95% CI: −1.48 to −0.45; *p* < 0.001), whereas it was +0.02 mL/min/year (−0.77 to +0.81; *p* = 0.96) in Eastern European-born KT recipients. There were no statistically significant differences in transplant survival beyond 1 year after KT [it was 91.8% in EU-born (95% CI: 87.1–96.8); 92.5% in Eastern European-born (86.1–99.4); and 89.3% in non-European-born recipients (83.0–96.0)].

#### Transplantation and Non-Refusal Rates to Deceased Organ Donation

Only one regional study from Piedmont assessed transplantation and non-refusal rates to deceased organ donation among immigrant family members in intensive care units (ICU) [51]. Between 2004 and 2011, out of 2,914 transplants, first-generation immigrants [*n* = 222 (7%)] received liver (*n* = 66), kidney (*n* = 130), heart (*n* = 21) and lung (*n* = 5) transplantation. Acceptance of deceased organ donation was lower in some ethnic groups, regardless of immigration background. Families originating from Romania had the most favorable attitude towards organ donation (78.8%), whereas families of Moroccan and Albanian origin were the least favorable (25% and 33%, respectively) [51]. These data were not adjusted for any potential confounding factors.

## Discussion

ODT is a complex process with many clinical, psychosocial, and cultural factors that may be associated with immigrant status. Patient and family education and informed consent are keys to equitable care for vulnerable populations throughout the ODT process. Shared decision-making is the most desirable and ethical model of informed consent at all stages of ODT [52] but may be impeded by multiple factors at the level of patients, providers, the clinical encounter, the healthcare system, and the broader societal context. “The ethical foundation of informed consent can be traced to the promotion of two values: personal wellbeing and self-determination.” This can be achieved only when the informed consent process is “based upon mutual respect and participation, not a ritual to be equated with reciting the contents of a form … ” [53]. Our study newly shows that, despite a publicly funded health system with universal healthcare coverage, non-European-born individuals living in Italy are less likely to receive LDKT and more likely to have inferior long-term kidney graft function compared with EU-born and Eastern European-born individuals. Further, while immigrant patients are increasingly represented among transplant recipients (especially kidney and liver transplants), refusal rates for organ donation are higher in some ethnic groups compared with native-born and other foreign-born referents. However, existing literature based on regional and national registries focuses on KT with descriptive analyses not adjusted for all potential confounding variables. Qualitative studies assessing the perspectives of relevant stakeholders, the lay public, bereaved family members, patients pursuing transplant, (potential) living donors from immigrant communities, and transplant centers and ICU healthcare professionals (HCP) are missing. Such studies would inform on the potential barriers to transplant care at the individual, interpersonal, and the societal level to identify potential areas for intervention [1]. In addition, evidence is lacking of the patient experience throughout the various stages of the ODT pathway (from end stage organ disease onset through to post-transplant follow-up). These studies could provide insights into the factors which may have negative downstream effects on the outcomes of transplantation for individuals with an immigration background. The higher refusal rates and reduced live donation among ethnic minority populations have the potential to increase the waiting time for transplantation [18, 54]. The inability to match donor and recipient ethnicity may negatively impact on patient and graft survival [55, 56].

Discrepancy between transplantation and refusal rates to organ donation [18, 19] are not indicative of a lack of reciprocity or unequal contribution to organ donation among immigrant populations. Rather, as for the other phases of the ODT process, they are likely a reflection of multiple, intersecting factors [17, 18, 33, 57–60]. Among immigrant populations, the lack of knowledge regarding ODT, varied cultural and religious beliefs, low health literacy, lack of trust towards the healthcare system and HCPs in the host country, a systemic inability to communicate about ODT in a culturally sensitive fashion, and the background framework of ODT in the country of origin with fear of organ trafficking, unfairness, lack of transparency, or general mistrust towards the healthcare system may contribute to negative attitudes towards organ donation in some immigrant groups [17, 18, 33, 57–60].

Studies from across Europe have drawn attention to the association of immigrant and/or ethnic minority status with inequities in ODT. An incident cohort study of 11,299 ESKD patients from the United Kingdom (UK) (9,602 White, 1,178 South Asian, 519 Black) [9] found that individuals of South Asian and Black ethnicity are younger, more socially deprived, and have more diabetes. Such individuals are more likely to be referred later to a nephrologist relative to White referents (*p* = 0.01). After adjusting for patient characteristics, social deprivation, and center effects, South Asian patients were more likely to be wait-listed compared with White patients, whereas no differences were noted between patients of Black and White ethnicity [9]. The ethnic composition of this patient cohort was different from that of Italian studies (i.e., in all three Italian studies on waitlisting, the immigrant category comprised patients of both White and non-White ethnicity), and no data were available of the patients’ immigration background or family history of migration. Besides, ethnic diversity presents substantial differences between Italy and the UK. Yet, the findings of delayed nephrological referral in the UK and Italy are similar. Despite the UK and Italy having a universal healthcare system, it is possible that the lower use of primary and specialist care among individuals from ethnic and immigrant minority communities may explain a portion of delayed referrals [26, 61]. WL accessibility is higher or equal in immigrant groups when compared with their non-immigrant counterparts in Lombardy and Lazio, respectively, but lower in Piedmont. Italy and the UK provide lifelong transplant care which may dampen the adverse effect of the patient’s insurance status or socioeconomic deprivation level on WL enrollment [62]. However, the Italian healthcare and welfare systems are decentralized, and each region is in charge of organizing and delivering healthcare, social care and social welfare services to the population [63]. Therefore, in Italy, systemic regional differences may account as potentially confounding factors and may explain a portion of existing differences among regions. Besides, although socioeconomic deprivation is common amongst immigrant communities in Italy [30], none of the studies included in this review explored the interplay between immigrant status, ethnicity and socioeconomic deprivation.

Other studies have noted the association of immigrant status and/or ethnicity with reduced likelihood of preemptive or LDKT [10, 12, 14, 15], although some - including the study included in this review—did not account for socioeconomic status [10, 49]. In the UK [12], socioeconomic deprivation (*p* < 0.001) and ethnicity (*p* = 0.005) are significant, independent and interactive predictors. This translates into a marked difference in the proportions of LDKTs between White and non-White recipients in the most socioeconomically deprived groups (39.5% versus 19.3%), but not among the least deprived segments (48.5% versus 51.9%) [12]. One single center retrospective cohort study of 77 pediatric patients [32 born of immigrant families (10 from former Yugoslavia, 10 from Turkey, 10 from other countries]) from Austria [64] contradicted this finding, despite the presence of multiple migration-related inequities (i.e., information delay, limited communication, low levels of knowledge, and self-reported conflicting beliefs) [64]. However, this was largely a White, Eastern European cohort with a longer standing immigration history [46]. Inferior likelihood of LDKT may result from the interplay among various factors at the level of patients, potential living donors, providers, and the healthcare system. These may include personal, religious or cultural beliefs, limited health literacy, lack of family members living close enough to be evaluated for donation, lack of support, inability to take time off work for potential living donors, inadequate communication, suboptimal patient/living donor education, providers’ biases or concerns about non-resident donors’ follow-up, and lack of healthcare policies for coverage of travel and medical fees for living-donor surgery and follow-up of non-resident donors [22, 24, 49, 65–71].

Most studies of the outcomes of transplantation among immigrants and/or ethnic minority communities [11, 15, 16, 72–76] find no statistically significant differences in patient or graft survival. The study by Grossi et al. included in this review is the only study which found an association between immigration background with worse kidney graft function decline following KT [50]. One single center retrospective cohort study of 555 (50 Black, 505 non-Black) KT recipients from the UK found that, independent of CYP3A5 expresser status, Black KT recipients have poorer long-term outcomes relative to those from other ethnic groups [16]. Similarly, a pediatric study from the Netherlands found higher rejection rates among immigrant KT recipients when compared to non-immigrant referents [10]. As access to care is provided in these regions, it is possible that inequities in earlier stages of transplant care play a role. Late referrals, inferior rates of preemptive and/or LDKT, inadequate patient education, and inability to address socioeconomic and cultural factors associated with immigrant status are some examples [15, 77]. Immigration may also contribute to different exposures to opportunistic infections. Ethnicity may be associated with higher alloreactive immune reactions or metabolism of immunosuppressive medications [10, 15, 75, 78]. The impacts of social determinants of health associated with immigration may also account for a portion of unfavorable trends in KT outcomes [31, 79].

### Interventions

Current approaches to communication, patient education and management are less likely to be effective with subjects from immigrant and/or ethnic minority groups. Targeted/tailored interventions to meet the needs of these populations remain limited in Europe. Studies have advocated for a coordinated approach to empower these individuals at all stages of the process by encouraging participation and inclusion [1]. Studies in the UK [59], Spain [80], and the Netherlands [81] have attempted to improve dialogue with diverse ethnic and faith communities to empower HCPs to deliver more culturally competent and family-centered approaches regarding organ donation and LDKT [57, 80]. Culturally and linguistically competent websites or transplant-center based education to empower specific ethnic minority groups by targeting their needs may also prove useful [82, 83].

**FIGURE 1:**
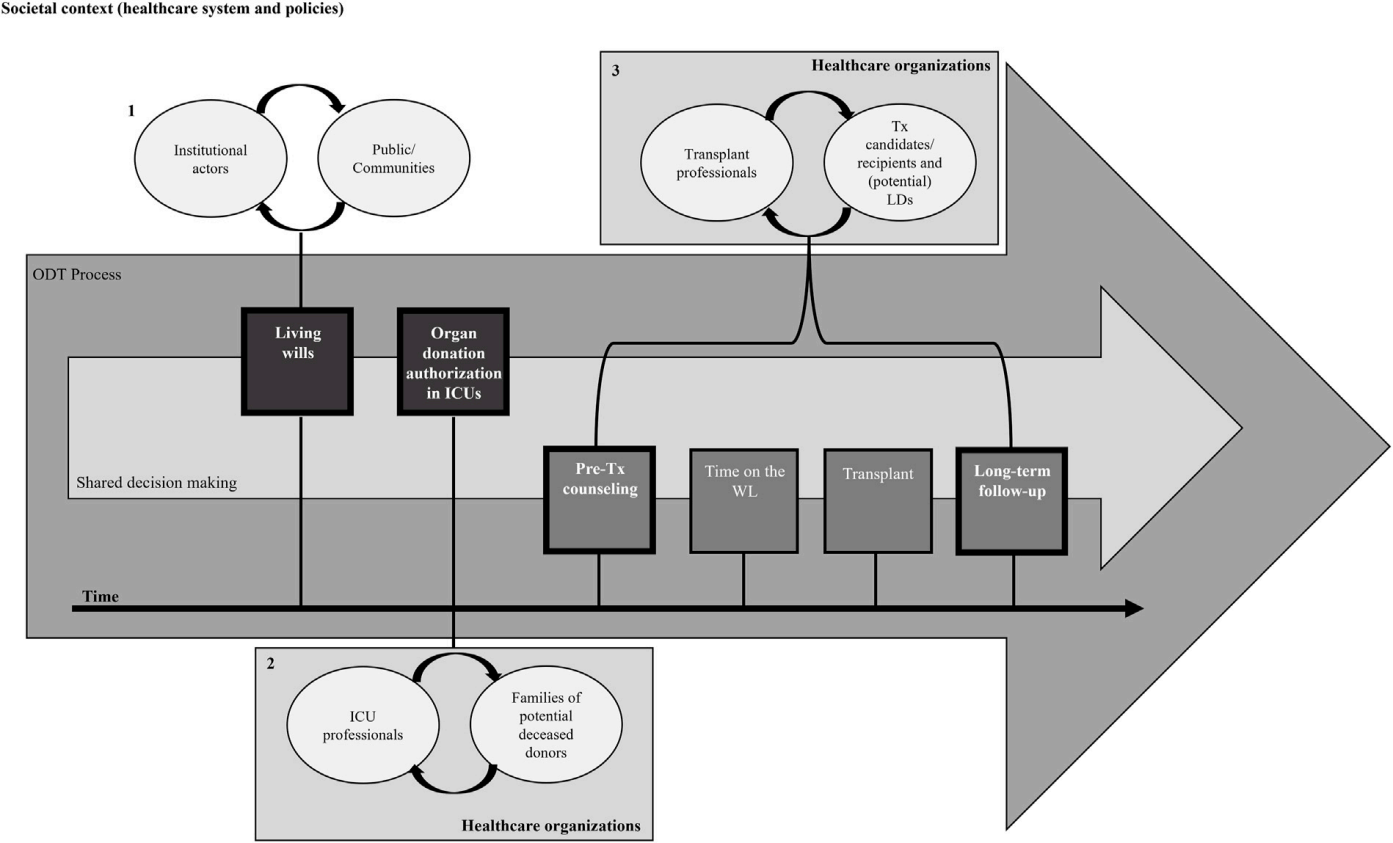
Conceptual model of the FAITH project of the Italian National Transplant Center. The figure illustrates the centrality of shared decision making at all stages of the organ donation and transplantation process. The first communication process refers to the relationship between institutional actors and the general public/communities (1), the second to the relationship between healthcare professionals in ICUs and the family members of potential deceased donors (2), and the third to the relationship between transplant professionals and transplant candidates/recipients/(potential) living donors (3). The figure also points out that, to enable the prevention of inequities, interventions should be implemented to target not only communities and/or individuals with an immigration background, but also those who relate with them, namely, institutional actors (1), ICU (2) and transplant centers’ HCPs (3), and the contexts where these processes respectively occur [i.e., the societal context (especially relative to the healthcare system—with a focus on healthcare organizations for stages 2 and 3—and policies)]. HCP, Healthcare Professionals; ICU, Intensive Care Unit; LD, Living Donor; MEM, Migrant and Ethnic Minority; Tx, Transplant; WL, Waiting List. *Adapted and modified from Grossi and Cardillo [86].

In Italy, multilingual donation-related informative material and courses on ODT for cultural mediators and other HCPs involved in organ procurement in some centers have improved the cultural appropriateness of end-of-life care [84, 85]. CNT has initiated a project titled “*Fostering And Improving equity, participation and inclusion in Transplantation Healthcare*” (FAITH) [86] consistent with ESOT policies [4] to adapt ODT communicative, educational and management processes to the needs of immigrant communities, families and individuals (Figure 1) (a more detailed description of the research protocol and agenda is reported in the Supplementary Box SA).

It is possible that gaps may persist because of inequalities at the broader societal level and biological variations in some ethnic groups. However, considering the portion of inequities engrained in modifiable factors is a compelling ethical duty.

### Limitations and Strengths

Studies of inequities in ODT among immigrant populations residing in Italy included individuals with extreme diversity of cultural, religious, social and immigration backgrounds. These are major limitations as these aspects may significantly bias the interpretation of the results and the potential for intervention. Besides, we acknowledge that there can be no “one size fits all approach” to immigrant populations. For instance, because there can be great within-group variation, future studies should collect data to enable better understanding of the many factors intersecting with immigration. Potentially confounding factors include, but are not limited to, socio-economic status, educational and occupational background, country of origin or descent, reasons for and circumstances of migration, time elapsed since immigration as a proxy for acculturation and integration, language proficiency, local integration policies, and other systemic features which may vary among regions. Another limitation lies in the small number, size, and limited quality of studies. Studies are focused only on ESKD and KT. Yet, this is consistent with prior reviews of inequities in organ transplantation [87]. None investigated possible differences in the need for organ transplants among resident versus more vulnerable immigrant groups (i.e., refugees, asylum seekers, undocumented immigrants). However, studies have shown that the need for KT in these vulnerable groups is infrequent in Europe [88], and most immigrants in Italy are regular residents (5.7 million regular versus 519,000 irregular migrants in 2021) [89]. Italian findings might not apply to immigrant populations residing in countries with a different healthcare and social security system, with longer standing immigration history, or to second- or third-generation immigrants (i.e., subjects who were native-born of foreign-born families). As noted in prior reports, consensus is lacking about the categorization of these populations in Europe [1, 45]. This applies also to the studies included in our review. Because ethnicity-related statistics are not allowed in the Italian Census, immigrant status is identified by surrogates like citizenship, place of birth, former citizenship for Italians, and citizenship of parents [37], challenging data interpretation, reporting, and comparisons. Within-country and cross-country comparisons are further challenged by the immigrant category not always distinguishing between individuals who have migrated from EU and non-EU countries, and between people from non-EU countries (i.e., Eastern European countries vs. other non-European/non-Western countries) and between ethnicities. However, the greatest strength of this work is that, when compared to prior evidence from the UK and the US that consider broader ethnicity or racial categories (i.e., Hispanic, Asian or Black) [18, 33], data from Italy may provide additional insights related to immigrant status and country of birth to provide better understanding of trends and target actions [18]. The Italian pathway against inequities in ODT provided in the Supplementary Appendix may inform future similar initiatives in other countries. The findings derived from the preliminary feedback of key stakeholders in the ODT process (Supplementary Tables S2, S3) may contribute to targeted interventions on modifiable barriers to meet the needs of immigrant populations and provide more equitable transplant care among individuals from these communities.

## Abbreviations

CNT, Italian National Transplant Center; ESKD, End Stage Kidney Disease; EU, European Union; HCP, Healthcare Professional; ICU, Intensive Care Unit; KT, Kidney Transplant; LDKT, Living Donor Kidney Transplant; ODT; Organ Donation and Transplantation; UK, United Kingdom; WL, waiting list.

## References

[1] Grossi AA Paredes D Palaniswami V Jansen N Picozzi M Randhawa G. ‘One Size Does Not Fit All’ in Organ Donation and Transplantation: Targeting and Tailoring Communication for Migrant and Ethnic Minority Populations. Commun Med (2023) 18(3):241–57. 10.1558/cam.21434 39916086

[2] Vanholder R Domínguez-Gil B Busic M Cortez-Pinto H Craig JC Jager KJ Organ Donation and Transplantation: A Multi-Stakeholder Call to Action. Nat Rev Nephrol (2021) 17(8):554–68. 10.1038/s41581-021-00425-3 33953367 PMC8097678

[3] European Kidney Health Alliance. A Shared Vision for Improving Organ Donation and Transplantation in the EU (2019). Available from: https://ekha.eu/blog/updated-final-version-of-the-joint-statement-on-organ-donation-and-transplantation-now-available/ (Accessed July 25, 2023).

[4] European Society of Organ Transplantation (ESOT). Tackling Inequalities in Organ Transplantation: A Pathway Forward (2022). Available from: https://esot.org/wp-content/uploads/2022/10/EM012518_ESOT_ActionDay_ThinkTankReport_2201005_v0-8_FH.pdf (Accessed January 18, 2023).

[5] European Commission. Competence Centre on Foresight. Developments and Forecasts on Increasing Significance of Migration (2018). [Internet] Available from: https://knowledge4policy.ec.europa.eu/foresight/topic/increasing-significance-migration/more-developments_en (Accessed March 15, 2023).

[6] Schoenmaker NJ Tromp WF van der Lee JH Adams B Bouts AH Collard L Disparities in Dialysis Treatment and Outcomes for Dutch and Belgian Children With Immigrant Parents. Pediatr Nephrol (2012) 27(8):1369–79. 10.1007/s00467-012-2135-7 22434424 PMC3382654

[7] Pruthi R Robb MWL Oniscu GC Tomson C Bradley A Forsythe JL Inequity in Access to Transplantation in the United Kingdom. Clin J Am Soc Nephrol (2020) 15(6):830–42. 10.2215/CJN.11460919 32467306 PMC7274279

[8] Cantrelle C Laurens C Luciolli E Loty B Tuppin P. Access to Kidney Transplantation in France of Non-French Patients and French Patients Living in Overseas Territories. Transplantation (2006) 81(8):1147–52. 10.1097/01.tp.0000205182.96861.3a 16641600

[9] Udayaraj U Ben-Shlomo Y Roderick P Casula A Dudley C Johnson R Social Deprivation, Ethnicity, and Access to the Deceased Donor Kidney Transplant Waiting List in England and Wales. Transplantation (2010) 90(3):279–85. 10.1097/TP.0b013e3181e346e3 20523276

[10] Tromp WF Cransberg K van der Lee JH Bouts AH Collard L Van Damme-Lombaerts R Fewer Pre-Emptive Renal Transplantations and More Rejections in Immigrant Children Compared to Native Dutch and Belgian Children. Nephrol Dial Transpl (2012) 27(6):2588–93. 10.1093/ndt/gfr628 22323533

[11] Tjaden LA Noordzij M Van Stralen KJ Kuehni CE Raes A Cornelissen EAM Racial Disparities in Access to and Outcomes of Kidney Transplantation in Children, Adolescents, and Young Adults: Results From the ESPN/ERA-EDTA (European Society of Pediatric Nephrology/European Renal Association-European Dialysis and Transplant Association) Registry. Am J Kidney Dis (2016) 67(2):293–301. 10.1053/j.ajkd.2015.09.023 26561356

[12] Khalil K Brotherton A Moore S Evison F Gallier S Hodson J Interaction Between Socioeconomic Deprivation and Ethnicity for Likelihood of Receiving Living-Donor Kidney Transplantation. BMC Nephrol (2022) 23(1):113–1. 10.1186/s12882-022-02742-6 35305568 PMC8934457

[13] Wu DA Robb ML Watson CJE Forsythe JLR Tomson CRV Cairns J Barriers to Living Donor Kidney Transplantation in the United Kingdom: A National Observational Study. Nephrol Dial Transpl (2017) 32(5):890–900. 10.1093/ndt/gfx036 PMC5427518 28379431

[14] Udayaraj U Ben-Shlomo Y Roderick P Casula A Dudley C Collett D Social Deprivation, Ethnicity, and Uptake of Living Kidney Donor Transplantation in the United Kingdom. Transplantation (2012) 93(6):610–6. 10.1097/TP.0b013e318245593f 22245879

[15] Laging M Kal-van Gestel JA van de Wetering J Ijzermans JNM Weimar W Roodnat JI. Understanding the Influence of Ethnicity and Socioeconomic Factors on Graft and Patient Survival After Kidney Transplantation. Transplantation (2014) 98(9):974–8. 10.1097/TP.0000000000000164 24926831

[16] Ng FL Holt DW Chang RWS MacPhee IAM. Black Renal Transplant Recipients Have Poorer Long-Term Graft Survival Than CYP3A5 Expressers From Other Ethnic Groups. Nephrol Dial Transpl (2010) 25(2):628–34. 10.1093/ndt/gfp530 19825838

[17] Ríos Zambudio A Carrillo J López-Navas AI Ayala-García MA Alconchel F Iniesta-Sepúlveda M African Immigrants Living in Spain: Awareness Toward Organ Donation and the Need for Specific Awareness Campaigns. Exp Clin Transpl (2022) 20(2):199–208. 10.6002/ect.2021.0480 35282812

[18] Randhawa G Gardiner D. Tackling Organ Donation Among Minority Ethnic Communities in the UK-A Whole Systems Approach. Br Med Bull (2022) 142(1):4–14. 10.1093/bmb/ldac008 35368069

[19] Bhopal A Wium C Reisæter AV Bhala N Kumar B. Organ Donation for Migrants and Ethnic Minorities. Tidsskr den Nor Laegeforening (2019) 139(13). 10.4045/tidsskr.19.0406 31556540

[20] Douglas P Cetron M Spiegel P. Definitions Matter: Migrants, Immigrants, Asylum Seekers and Refugees. J Trav Med (2019) 26(2):taz005. 10.1093/jtm/taz005 30753575

[21] Wickramage K Vearey J Zwi AB Robinson C Knipper M. Migration and Health: A Global Public Health Research Priority. BMC Public Health (2018) 18(1):987. 10.1186/s12889-018-5932-5 30089475 PMC6083569

[22] Poulakou G Len O Akova M. Immigrants as Donors and Transplant Recipients: Specific Considerations. Intensive Care Med (2019) 45(3):401–3. 10.1007/s00134-019-05534-z 30701293

[23] Chiarenza A Dauvrin M Chiesa V Baatout S Verrept H. Supporting Access to Healthcare for Refugees and Migrants in European Countries Under Particular Migratory Pressure. BMC Health Serv Res (2019) 19(1):513. 10.1186/s12913-019-4353-1 31337406 PMC6651950

[24] Van Biesen W Vanholder R Ernandez T Drewniak D Luyckx V. Caring for Migrants and Refugees With End-Stage Kidney Disease in Europe. Am J Kidney Dis (2018) 71(5):701–9. 10.1053/j.ajkd.2017.10.015 29274918

[25] Sever MŞ Jager KJ Vanholder R Stengel B Harambat J Finne P A Roadmap for Optimizing Chronic Kidney Disease Patient Care and Patient-Oriented Research in the Eastern European Nephrology Community. Clin Kidney J (2021) 14(1):23–35. 10.1093/ckj/sfaa218 33570513 PMC7857792

[26] Di Napoli A Ventura M Spadea T Giorgi Rossi P Bartolini L Battisti L Barriers to Accessing Primary Care and Appropriateness of Healthcare Among Immigrants in Italy. Front Public Heal (2022) 10:817696. 10.3389/fpubh.2022.817696 PMC8864157 35223739

[27] Ballotari P Caroli S Ferrari F Romani G Marina G Chiarenza A Differences in Diabetes Prevalence and Inequalities in Disease Management and Glycaemic Control by Immigrant Status: A Population-Based Study (Italy). BMC Public Health (2015) 15:87. 10.1186/s12889-015-1403-4 25884923 PMC4334763

[28] Eurostat. Migrant Population: 23.7 Million Non-EU Citizens Living in the EU on 1 January 2021 (2022). [Internet] Available from: https://ec.europa.eu/eurostat/statistics-explained/index.php?title=Migration_and_migrant_population_statistics#Migrant_population:_23.7_million_non-EU_citizens_living_in_the_EU_on_1_January_2021 (Accessed December 1, 2022).

[29] Italian National Institutes of Statistics (ISTAT). Identità e Percorsi di Integrazione Delle Seconde Generazioni in Italia. Rome (2020). Available from: https://www.istat.it/it/archivio/240930 (Accessed July 25, 2023).

[30] ISMU. The Twenty-Fourth Italian Report on Migrations 2018 (2019). Fondazione ISMU Available from: https://www.ismu.org/en/the-twenty-fourth-italian-report-on-migrations-2018/ (Accessed March 10, 2023).

[31] Wändell P Carlsson AC Li X Gasevic D Ärnlöv J Sundquist J End-Stage Kidney Diseases in Immigrant Groups: A Nationwide Cohort Study in Sweden. Am J Nephrol (2019) 49(3):186–92. 10.1159/000497063 30712037 PMC6433394

[32] Hounkpatin HO Fraser SDS Honney R Dreyer G Brettle A Roderick PJ. Ethnic Minority Disparities in Progression and Mortality of Pre-Dialysis Chronic Kidney Disease: A Systematic Scoping Review. BMC Nephrol (2020) 21(1):217–4. 10.1186/s12882-020-01852-3 32517714 PMC7282112

[33] Bratton C Chavin K Baliga P. Racial Disparities in Organ Donation and Why. Curr Opin Organ Transpl (2011) 16(2):243–9. 10.1097/MOT.0b013e3283447b1c 21415828

[34] Di Napoli A Lapucci E Baglio G Di Giulio S, Registro Regionale Dialisi e Trapianto del. Lazio Dialysis Registry: Natives vs Foreigners. G Ital Di Nefrol (2015) 32(3):gin/32.3.8. 26093137

[35] Forneris G Boero R Massara C Quarello F. Immigrants and Dialysis: A Survey in Piedmont. G Ital Di Nefrol (2011) 28(3):314–8. 21626500

[36] Corghi E Conte F Esposito V Galassi A Galli EG La Rocca E Non-EU Patients in Dialysis Centers in Lombardy [I Pazienti Extracomunitari Nei Centri Dialisi Della Lombardia]. G Ital Di Nefrol (2016) 33(67).

[37] Affronti M Affronti A Soresi M Giannitrapani L Campagna E Tramuto F Distribution of Liver Disease in a Cohort of Immigrants in Sicily: Analysis of Day-Hospital Admissions in a Migration Medicine Unit. Le Infez Med (2014) 3:200–5. 25269961

[38] Buja A Gini R Visca M Damiani G Federico B Francesconi P Prevalence of Chronic Diseases by Immigrant Status and Disparities in Chronic Disease Management in Immigrants: A Population-Based Cohort Study, Valore Project. BMC Public Health (2013) 13:504. 10.1186/1471-2458-13-504 23706129 PMC3673842

[39] Gibertoni D Mammana L Gherardi G Baschieri E Minora F Santoro A. Presentation and Outcome of Chronic Kidney Disease in Italian and Immigrant Citizens: Results From the Emilia-Romagna PIRP Project. J Nephrol (2022) 35(1):179–90. 10.1007/s40620-021-00984-5 33595822

[40] National Academies of Sciences, Engineering and Medicine. Confronting and Eliminating Inequities in the Organ Transplantation System. In: Hackmann M English R Kizer K, editors. Realizing the Promise of Equity in the Organ Transplantation System. Washington, DC: The National Academies Press (2022). p. 85–115. 35226429

[41] Braveman P. Health Disparities and Health Equity: Concepts and Measurement. Annu Rev Public Health (2006) 27:167–94. 10.1146/annurev.publhealth.27.021405.102103 16533114

[42] International Organization for Migration (IOM). Glossary on Migration (2019). Available from: https://publications.iom.int/system/files/pdf/iml_34_glossary.pdf (Accessed January 10, 2023).

[43] European Commission. “Migrant” Definition (2023). [Internet]. Available from: https://home-affairs.ec.europa.eu/pages/glossary/migrant_en (Accessed January 10, 2023).

[44] Bhopal R. Glossary of Terms Relating to Ethnicity and Race: For Reflection and Debate. J Epidemiol Community Health (2004) 58(6):441–5. 10.1136/jech.2003.013466 15143107 PMC1732794

[45] Grossi AA Randhawa G Jansen NE Paredes D. Taking a “Care Pathway/whole Systems” Approach to Equality Diversity Inclusion (EDI) in Organ Donation and Transplantation in Relation to the Needs of “Ethnic/racial/migrant” Minority Communities:A Statement and a Call for Action. Transpl Int (2023) 36. 10.3389/ti.2023.11310 PMC10437067 37600748

[46] EUPHA. Migration, Ethnicity and Health. Statement Drafted by the Section for Migration, Ethnicity and Health of the European Public Health Association (EUPHA) (2018). Available from: https://ephconference.eu/repository/sections/migr/EUPHA_statement_9th_May_2018a_revised.pdf (Accessed March 3, 2023).

[47] Paglialonga F Consolo S Vidal E Parolin M Minale B Giordano M Resident Foreign Patients Receive Adequate Dialysis but Fewer Preemptive Transplantations: Data From the Italian Pediatric Dialysis Registry. Pediatr Nephrol (2021) 36(3):639–47. 10.1007/s00467-020-04730-0 32914248

[48] Fogazzi GB Castelnovo C. Maintenance Dialysis in Patients From Developing Countries: The Experience of an Italian Center. J Nephrol (2004) 17(4):552–8. 15372418

[49] Grossi AA Puoti F Fiaschetti P Di Ciaccio P Maggiore U Cardillo M. Kidney Transplantation and Withdrawal Rates Among Wait-Listed First-Generation Immigrants in Italy. Eur J Public Health (2022) 32(3):372–8. 10.1093/eurpub/ckac027 35381065 PMC9159323

[50] Grossi AA Maggiore U Puoti F Grossi PA Picozzi M Cardillo M. Association of Immigration Background With Kidney Graft Function in a Publicly Funded Health System: A Nationwide Retrospective Cohort Study in Italy. Transpl Int (2020) 33(11):1405–16. 10.1111/tri.13688 32621764

[51] Guermani A Potenza R Isnardi D Peluso M Bosco R Donadio P. Organ Donation and Transplantation in Migrants: Piedmont Reality From 2004 to 2011. Transpl Proc (2013) 45(7):2591–3. 10.1016/j.transproceed.2013.07.031 24033998

[52] Suurmond J Seeleman C. Shared Decision-Making in an Intercultural Context: Barriers in the Interaction Between Physicians and Immigrant Patients. Patient Educ Couns (2006) 60(2):253–9. 10.1016/j.pec.2005.01.012 16442467

[53] Michigan Law Review. Making Health Care Decisions: A Report on the Ethical and Legal Implications of Informed Consent in the Patient-Practitioner Relationship: Volume One. Mich L Rev (1984) 82(4):839–42. Available from: https://repository.law.umich.edu/mlr/vol82/iss4/21/ (Accessed January 11, 2023).

[54] Morgan M Sims J Jain N Randhawa G Sharma S Kirit M. Who Waits Longest for a Kidney?: Inequalities in Access to Kidney Transplantation Among Black and Asian Minority Ethnic (BAME) Groups in the UK. Br J Ren Med (2015) 20(1):4–7.

[55] Kanter KR Berg AM Mahle WT Vincent RN Kilgo PD Kogon BE Donor-Recipient Race Mismatch and Graft Survival After Pediatric Heart Transplantation. Ann Thorac Surg (2009) 87(1):204–9. 10.1016/j.athoracsur.2008.09.074 19101298

[56] Allen JG Weiss ES Merlo CA Baumgartner WA Conte JV Shah AS. Impact of Donor-Recipient Race Matching on Survival After Lung Transplantation: Analysis of Over 11,000 Patients. J Hear Lung Transpl (2009) 28(10):1063–71. 10.1016/j.healun.2009.06.012 19782288

[57] Morgan M Kenten C Deedat S Farsides B Newton T Randhawa G Increasing the Acceptability and Rates of Organ Donation Among Minority Ethnic Groups: A Programme of Observational and Evaluative Research on Donation, Transplantation and Ethnicity (DonaTE). Program Grants Appl Res (2016) 4(4):1–196. 10.3310/pgfar04040 27054221

[58] Kentish-Barnes N Siminoff LA Walker W Urbanski M Charpentier J Thuong M A Narrative Review of Family Members’ Experience of Organ Donation Request After Brain Death in the Critical Care Setting. Intensive Care Med (2019) 45(3):331–42. 10.1007/s00134-019-05575-4 30840119

[59] Randhawa G Neuberger J. Role of Religion in Organ Donation-Development of the United Kingdom Faith and Organ Donation Action Plan. Transpl Proc (2016) 48(3):689–94. 10.1016/j.transproceed.2015.10.074 27234715

[60] El Hangouche AJ Alaika O Rkain H Najdi A Errguig L Doghmi N Knowledge, Attitudes, and Practice of Organ Donation in Morocco: A Cross-Sectional Survey. Saudi J Kidney Dis Transpl (2018) 29(6):1358–65. 10.4103/1319-2442.248301 30588967

[61] Lebano A Hamed S Bradby H Gil-Salmerón A Durá-Ferrandis E Garcés-Ferrer J Migrants’ and Refugees’ Health Status and Healthcare in Europe: A Scoping Literature Review. BMC Public Health (2020) 20(1):1039–22. 10.1186/s12889-020-08749-8 32605605 PMC7329528

[62] Zhang X Melanson TA Plantinga LC Basu M Pastan SO Mohan S Racial/ethnic Disparities in Waitlisting for Deceased Donor Kidney Transplantation 1 Year After Implementation of the New National Kidney Allocation System. Am J Transpl (2018) 18(8):1936–46. 10.1111/ajt.14748 PMC6105401 29603644

[63] de Belvis A Meragaglia M Morsella A Adduci A Perilli A Cascini F Italy: Health System Review. Vol. 24, Health Systems in Transition (2022). Available from: https://eurohealthobservatory.who.int/publications/i/italy-health-system-review-2022 (Accessed July 18, 2023). 36951263

[64] Oztek-Celebi FZ Herle M Ritschl V Kaltenegger L Stamm T Aufricht C High Rate of Living Kidney Donation to Immigrant Children Despite Disparities- An Epidemiological Paradox? Front Pediatr (2019) 7:25. 10.3389/fped.2019.00025 30809513 PMC6379308

[65] Sandal S Charlebois K Fiore JF Wright DK Fortin MC Feldman LS Health Professional–Identified Barriers to Living Donor Kidney Transplantation: A Qualitative Study. Can J Kidney Heal Dis (2019) 6:2054358119828389. 10.1177/2054358119828389 PMC6376531 30792874

[66] Ismail SY Massey EK Luchtenburg AE Claassens L Zuidema WC Busschbach JJV Religious Attitudes Towards Living Kidney Donation Among Dutch Renal Patients. Med Health Care Philos (2012) 15(2):221–7. 10.1007/s11019-011-9326-z 21512856 PMC3319887

[67] Timmerman L Ismail SY Luchtenburg AE Zuidema WC Ijzermans JNM Busschbach JJV Exploring Knowledge About Dialysis, Transplantation, and Living Donation Among Patients and Their Living Kidney Donors. Int J Behav Med (2015) 22(5):580–9. 10.1007/s12529-015-9461-7 25634574 PMC4577545

[68] Ismail SY Claassens L Luchtenburg AE Roodnat JI Zuidema WC Weimar W Living Donor Kidney Transplantation Among Ethnic Minorities in the Netherlands: A Model for Breaking the Hurdles. Patient Educ Couns (2013) 90(1):118–24. 10.1016/j.pec.2012.08.004 22940372

[69] Taylor DM Bradley JA Bradley C Draper H Dudley C Fogarty D Limited Health Literacy Is Associated With Reduced Access to Kidney Transplantation. Kidney Int (2019) 95(5):1244–52. 10.1016/j.kint.2018.12.021 30952457

[70] Ladin K Rodrigue JR Hanto DW. Framing Disparities Along the Continuum of Care From Chronic Kidney Disease to Transplantation: Barriers and Interventions. Am J Transpl (2009) 9(4):669–74. 10.1111/j.1600-6143.2009.02561.x PMC2697924 19344460

[71] Massie AB Muzaale AD Luo X Chow EKH Locke JE Nguyen AQ Quantifying Postdonation Risk of ESRD in Living Kidney Donors. J Am Soc Nephrol (2017) 28(9):2749–55. 10.1681/ASN.2016101084 28450534 PMC5576930

[72] Bucher JN Koenig M Schoenberg MB Thomas M Crispin A Angele MK Liver Transplantation in Patients With a History of Migration-A German Single Center Comparative Analysis. PLoS One (2019) 14(10):e0224116. 10.1371/journal.pone.0224116 31639158 PMC6804963

[73] Oztek FZ Tekin P Herle M Mueller T Arbeiter K Aufricht C. Does Immigration Background Influence Outcomes After Renal Transplantation? Pediatr Nephrol (2011) 26(2):309–15. 10.1007/s00467-010-1685-9 21052728

[74] Oztek FZ Ipsiroglu O Mueller T Aufricht C. Outcome After Renal Transplantation in Children From Native and Immigrant Families in Austria. Eur J Pediatr (2009) 168(1):11–6. 10.1007/s00431-008-0698-x 18351389

[75] Mérida E Rodríguez A Hernandez G Huerta A Gonzalez J Hernández A Renal Transplantation in Emigrants From Africa in Spain: Similar Results but Different Infectious Profile Compared With Spanish People. Transpl Proc (2009) 41(6):2363–5. 10.1016/j.transproceed.2009.05.005 19715920

[76] Pallet N Thervet E Alberti C Emal-Aglae V Bedrossian J Martinez F Kidney Transplant in Black Recipients: Are African Europeans Different From African Americans. Am J Transpl (2005) 5(11):2682–7. 10.1111/j.1600-6143.2005.01057.x 16212627

[77] Skelton SL Waterman AD Davis LSA Peipert JD Fish AF. Applying Best Practices to Designing Patient Education for Patients With End-Stage Renal Disease Pursuing Kidney Transplant. Prog Transpl (2015) 25(1):77–84. 10.7182/pit2015415 PMC4489708 25758805

[78] Gordon EJ Ladner DP Caicedo JC Franklin J. Disparities in Kidney Transplant Outcomes: A Review. Semin Nephrol (2010) 30(1):81–9. 10.1016/j.semnephrol.2009.10.009 20116652 PMC2818243

[79] Campostrini S Carrozzi G Severoni S Masocco M Salmaso S Balestra F Migrant Health in Italy: A Better Health Status Difficult to Maintain - Country of Origin and Assimilation Effects Studied From the Italian Risk Factor Surveillance Data. Popul Health Metr (2019) 17(1):14–1. 10.1186/s12963-019-0194-8 31675961 PMC6824084

[80] Generalitat de Catalunya. Donació I Religions (2022). [Internet]. Available from: https://trasplantaments.gencat.cat/ca/la_donacio/donacio-i-religions/ (Accessed January 15, 2023).

[81] Ismail SY Luchtenburg AE Timman R Zuidema WC Boonstra C Weimar W Home-Based Family Intervention Increases Knowledge, Communication and Living Donation Rates: A Randomized Controlled Trial. Am J Transpl (2014) 14(8):1862–9. 10.1111/ajt.12751 24935081

[82] Gordon EJ Feinglass J Carney P Vera K Olivero M Black A A Culturally Targeted Website for Hispanics/latinos About Living Kidney Donation and Transplantation: A Randomized Controlled Trial of Increased Knowledge. Transplantation (2016) 100(5):1149–60. 10.1097/TP.0000000000000932 26444846

[83] Gordon EJ Caicedo JC Ladner DP Reddy E Abecassis MM. Transplant Center Provision of Education and Culturally and Linguistically Competent Care: A National Study. Am J Transpl (2010) 10(12):2701–7. 10.1111/j.1600-6143.2010.03304.x 21158005

[84] Potenza R Guermani A Peluso M Casciola A Ginosa I Sperlinga R Effectiveness of an Education Program on Donation and Transplant Aimed at Students of the Nursing Degree Course. Transpl Proc (2015) 47(7):2097–101. 10.1016/j.transproceed.2014.11.074 26361652

[85] Potenza R Guermani A Grosso M Fossarello L Fontaneto C Casciola A Organ and Tissue Donation in Migrants: Advanced Course for Cross-Cultural Mediators. Transpl Proc (2013) 45(7):2584–6. 10.1016/j.transproceed.2013.07.029 24033996

[86] Grossi AA Cardillo M. Il Progetto Migrant and Ethnic Minority Education on Transplantation and Organ Donation and Process Optimization (ME TOO). Trapianti (2021) 25(3):73–85. 10.1709/3687.36736

[87] Park C Jones MM Kaplan S Koller FL Wilder JM Boulware LE A Scoping Review of Inequities in Access to Organ Transplant in the United States. Int J Equity Health (2022) 21(1):22–0. 10.1186/s12939-021-01616-x 35151327 PMC8841123

[88] Van Biesen W Vanholder R Vanderhaegen B Lameire N Wanner C Wiecek A Renal Replacement Therapy for Refugees With End-Stage Kidney Disease: An International Survey of the Nephrological Community. Kidney Int Suppl (2016) 6(2):35–41. 10.1016/j.kisu.2016.09.001 PMC6340918 30675418

[89] ISMU (Iniziative e Studi sulla MUltietnicità). XXVII Rapporto Sulle Migrazioni 2021 (2021). [XXVII Migration report 2021]. Available from: https://www.ismu.org/xxvii-rapporto-sulle-migrazioni-2021-comunicato-stampa-11-2-2022/ (Accessed July 20, 2023).

