# Supplementary material for: Inequities in Organ Donation and Transplantation Among Immigrant Populations in Italy: A Narrative Review of Evidence, Gaps in Research and Potential Areas for Intervention
Source: Transpl Int. 2023 Aug 11;36:11216. doi: 10.3389/ti.2023.11216 (PMC10450150; doi:10.3389/ti.2023.11216)
Supplement: Supplementary file 1 [file DataSheet1.pdf]

Supplementary Appendix

Suppl. Box A. Detailed description of the FAITH Project protocol

Rationale
The rationale for the FAITH project lies in what has been presented in the review work.

Objectives
This study aims to determine the informative, cultural, and psychosocial needs of immigrant populations and individuals in relation to the entire ODT process, with the ultimate goal of promoting informative, decision-making, relational, and management processes that are truly informed and tailored to the specific needs of immigrant populations.

The specific objectives are:

(i) To explore what factors at the individual, interpersonal, and societal levels – as well as the interaction among the different levels – may affect the intention and the subsequent act of manifesting a favorable living will about organ donation among different ethnic and faith communities.

(ii) To deliver information about ODT to the public/communities in a culturally sensitive fashion.

(iii) To assess ICU healthcare professionals (HCP) (inter)cultural competences (awareness, sensitivity, knowledge, attitudes, family-centered communication, behaviors), relational difficulties during donation interviews with the bereaved family members of immigrant patients, the *diversity-responsiveness* of local healthcare organizations (i.e., the ability of health systems and healthcare organizations to respond to the needs of a diverse population) (1), and the usefulness of initiatives to enhance these processes.

(iv) To assess the features of the transplant education process and follow-up management of immigrant patients, determine the (inter)cultural competence of transplant HCPs, including healthcare practices and organizational features (i.e., *diversity responsiveness*), the problems most frequently encountered by transplant centers' HCPs, and the usefulness of possible initiatives to improve these processes.

(v) To deliver relevant information about the specific needs of immigrant bereaved donors' families to ICU HCPs, and about the main obstacles to patient education and management in relation to transplantation for transplant centers' HCPs

Methods and analyses
Stakeholder involvement
The CNT established a permanent working group including representatives of the key stakeholders in the ODT process across the regions participating in the project: regional and local transplant coordinators, ICU specialists, transplant center nurses, psychologists, social workers, cultural mediators, patient and donor associations, gender-specific medicine experts, foreign-born physicians, transplant recipients with an immigration background, communication and medical ethics experts, education specialists, migration medicine and migrants/refugees' assistance experts, and religious communities. All key stakeholders were involved in the study design (see below) since the project start. Multiple meetings were held with stakeholder representatives to enable discussions surrounding the project objectives and its ethical implications (2,3), elicit their perspectives on the potential barriers to communication, education and management, gain insights on how to enable shared decision-making at the different stages of the ODT process, and discuss about earlier national and international experiences of targeted and/or tailored interventions directed towards immigrant populations and individuals, which were reviewed prior to the project start (3).

Study design

This study adopts a cross-sectional approach to pursue the study objectives, and a descriptive, multicenter, nonrandomized, prospective design. The study is based chiefly on self-administered questionnaires and will be performed over a 2-year period (01/05/2023-30/04/2025) including four phases: start-up (3 months), enrollment (8 months), data analyses (6 months), and dissemination of study results (4 months).

Three distinct questionnaires were adapted and/or developed for subsequent administration to (a) ethnic and faith communities, (b) ICU, and (c) transplant centers' HCPs, respectively. More specifically, a previously validated questionnaire to explore the factors with the potential to affect decision-making about organ donation (4–6) – and empirically test the relations among them – for future administration to immigrant and native-born communities was adapted based on a review of the literature (3), the stakeholder's feedback and various multi-stakeholder group discussions to further customize it to the specific features of the Italian context and the target populations. Similarly, based on earlier models of cultural competence in healthcare (7,8), the questionnaires directed towards ICU/local procurement coordination centers' and transplant centers' HCPs were developed based on the relevant literature (3,9–11), stakeholders' feedback and multi-stakeholder group conversations (the qualitative analysis of the stakeholders' feedback regarding the factors with the potential to intervene at the individual, interpersonal, and societal levels at the three stages of the ODT process [these were categorized based on the socio-ecological model (12)], and the potential for intervention are reported in **Supplementary Table S2** and **Supplementary Table S3**).

Definitions

'*Migrant'*: "any person who is outside a State of which he or she is a citizen or national, or, in the case of a stateless person, his or her State of birth or habitual residence. The term includes migrants who intend to move permanently or temporarily, and those who move in a regular or documented manner as well as migrants in irregular situations" (13). The immigrant category excludes persons who travel for tourism or business purposes and excludes intra-EU mobility (14).

'*Ethnicity*': "the social group a person belongs to, and either identifies with or is identified with by others, as a result of a mix of cultural and other factors including language, diet, religion, ancestry, and physical features" that are shared by individuals in the same group (15).

As recommended by prior reports (16), both the features related to 'ethnicity' (including country of origin or descent) and 'immigrant status' (or family history of immigration) should be considered for the purposes of studies in relation to ODT (16). For instance, the European Public Health Association contends that, although immigration includes also elements of ethnicity, 'visible minorities' are likely to experience more significant inequities relative to their 'White' referents, similar to immigrants (17).

Study settings

The Italian National Health System is a regionally based system presenting variations across regions which may impact on various levels of healthcare delivery depending on resource availability and organizational standards. The study will therefore be performed across eight regions representative of the Northern (Lombardy, Piedmont, Veneto, Friuli Venezia-Giulia), Central (Emilia-Romagna, Tuscany, Lazio) and Southern (Sicily) Italian areas (the overall presence of foreign citizens in the regions participating in the project is reported in **Supplementary Figure S1** and the distribution of the most represented populations at the national and regional levels in **Supplementary Table S1**).

Study population

The study population will comprise three distinct groups, namely (a) ethnic and faith communities (including native-born populations, i.e., individuals who are native-born of at least one native-born parent), (b) ICU, and (c) transplant centers' HCPs (physicians, nurses, health and social care workers, psychologists, social workers, cultural mediators, volunteers and experts in bioethics/clinical ethics).

Inclusion and exclusion criteria

Inclusion criteria

For ethnic and faith communities: individuals aged ≥18 years, residing in the regions involved in the project, having the ability to understand the informative sheet and providing formal consent to processing personal data.

For healthcare professionals: all individuals working in the ICUs and/or local hospital coordination centers and transplant centers in the regions involved in the project (physicians, nurses, health and social care workers,

psychologists, social workers, cultural mediators, volunteers and experts in bioethics/clinical ethics) and providing formal consent to processing personal data will be recruited.

Exclusion criteria

All subjects who meet the inclusion criteria but are unable to understand the informative sheet, do not agree to participate in the study, or do not consent to processing personal data.

Sample size determination

1. Ethnic and faith communities

Given the composition of the ethnic and faith communities in the regions involved in the study (the size of the populations based on citizenship are reported in **Supplementary Table S1**), the sample size is 1,500 questionnaires for a 95% confidence interval and a 5% error limit, with reference to the variability of responses, since unknown, maximum possible and equal to 50%. Thus, assuming a collection of 200-300 questionnaires from native-born populations, the remaining 1,200-1,300 questionnaires will be collected from communities with an immigration background; the expected sample size contains a correction with reference to the proportion (expected <10%) of questionnaires with some incomplete responses (<15%).

(Our total population is the unknown n (for the regions considered)

- Assumed frequency (%) of the outcome factor in the population (p): 50% (thus it is assumed the greatest response variability, that is used precisely when the response in the population is unknown)

- Confidence limits as % of 100 (absolute +/- %): 5%
- Level of error: Ɛ=5%

Equation [as derived from Schaeffer et al. (18)]:

$n= [(Z^2_{1-\alpha/2}{*}p(1-p)/\varepsilon^2)] / [1+1/N *(Z^2_{1-\alpha/2} *p*(1-p)/ \varepsilon^2)]$

where:

n=sample size

N=population size

p=the estimated proportion

Ɛ=desired absolute precision or absolute level of precision

$Z^2\alpha/2$ = the critical value of the Normal distribution at α/2 (e.g. for a confidence level of 95%, α is 0.05 and the critical value is 1.96)

- Correction for Finite Population : n_cfp=n/(1+(n-1)/N)

2. ICU healthcare professionals

The total number of ICUs in the participating regions is 272. For a 95% confidence interval and an error limit of 5%, with reference to the variability of responses, since unknown, maximum possible and equal to 50% . Based on earlier studies performed with these facilities, and the direct knowledge of the network, an adherence of at least 80% of ICUs is expected. With a minimum of 3 HCPs completing the questionnaire per facility, the expected sample is approximately 800 questionnaires. However, considering the objective of HCPs' training, no constraints will be placed on the maximum number of adherences, which therefore could exceed the number indicated.

**Region**	**ICU count**	**First stage sampling (number of facilities) (α=0.05; Ɛ=0.1)**	**Overall questionnaires K=3**
PIEMONTE	35	**3**	**105**
LOMBARDIA	66	**3**	**198**
VENETO	36	**3**	**108**
FRIULI VENEZIA GIULIA	10	**3**	**30**
EMILIA ROMAGNA	29	**3**	**87**
TOSCANA	30	**3**	**90**
LAZIO	34	**3**	**102**

SICILIA	32	**3**	**96**
**Total**	**272**	**30**	**≈ 800**

3. Transplant centers' healthcare professionals
The total number of transplant centers is 70. Estimating an involvement of all facilities, and assuming the completion of at least 5 questionnaires at each center, the expected sample size is 280-320 questionnaires.

Region	Tx center count	First stage sampling (number of facilities) (α=0.05; Ɛ=0.1)	Overall questionnaires K=5
PIEMONTE	7	**5**	**35**
LOMBARDIA	16	**5**	**80**
VENETO	10	**5**	**50**
FRIULI VENEZIA GIULIA	3	**5**	**15**
EMILIA ROMAGNA	7	**5**	**35**
TOSCANA	6	**5**	**30**
LAZIO	14	**5**	**70**
SICILIA	7	**5**	**35**
**Total**	**70**	**40**	**280-320**

Variables and measurements
Socio-demographic characteristics
For ethnic and faith communities, socio-demographic data including age group, place of birth, parents' place of birth (if born abroad), respondent's and/or parents' (if born abroad) time elapsed since immigration, gender, years of schooling, occupational category, marital status, presence of children, religious/spiritual faith, and frequency of participation in the life of one's religious community will be collected. As for HCPs, the socio-demographic characteristics collected will include gender, age group, place of birth, nationality, religious/spiritual faith, participation in religious activities, profession, time spent working in the ICU/in transplantation, participation in educational courses for the development of (inter)cultural competences, and prior professional experience in developing countries. For transplant centers' HCPs, the indicative number of immigrant transplant candidates evaluated over the past 5 years, the problems most frequently affecting the time for wait-listing and follow-up management (ranking), will also be assessed.

1. Ethnic and faith communities
Factors influencing living will manifestation to be an organ donor
Knowledge (8 items) will be assessed using a *yes* and *no* scale. Attitude (4 items), beliefs (2 items), descriptive norms (1 item), perceived control (2 items), distrust towards caregivers (1 item), body integrity (2 items), trait transfer (2 items), perceived benefits (2 items), ick factors (2 items), jinx factors (1 item), religious beliefs (2 items), and subjective norms (2 items) will be measured on a 5-point Likert scale (*1 = strongly disagree, 2 = disagree, 3 = neither disagree nor agree, 4 = agree, 5 = strongly agree*). Participants will also be asked about the interpersonal and mass media source(s) from which, if so, they have obtained information about organ donation. The reason underlying the act of not having yet expressed one's opinion about organ donation (if applicable), will be assessed through a multiple-choice question. Two open-ended qualitative questions will finally serve to acquire additional comments surrounding ODT and suggestions to improve the cultural appropriateness of future informative initiatives.

2. ICU healthcare professionals
The data collected about the specific hospital/healthcare organization and ICU (10 items) will include the location (i.e., region), number of beds, patients' type (adult/pediatric), presence/absence of a neurosurgery unit and transplant center, indicative share of immigrant patients admitted to the ICU and of immigrant donors over the past 5 years, most frequent reason for organ donation refusal, residency of family members during donation interview (Italy/foreign country). The following dimensions will be measured on 5-point Likert scales: (Inter)cultural awareness, sensitivity, knowledge and attitudes (11 items) (*1 = strongly disagree, 2 = disagree, 3 = neither disagree nor agree, 4 = agree, 5 = strongly agree*); Practices and behaviors (17 items) and Family-centered communication (3 items) (1 = n*ever, 2 = rarely, 3 =*

sometimes, 4 = often, 5 = always), and Usefulness of specific interventions (9 items) (*1 = completely useless, 2 = somewhat useless, 3 = uncertain, 4 = somewhat useful, 5 = very useful*). Two open-ended qualitative questions will finally serve to collect additional comments and suggestions about the difficulties encountered during donation interviews with the family members of potential deceased donors with an immigration background and other possible interventions to improve relational processes in ICUs.

3. Transplant centers' healthcare professionals

The data collected about the specific hospital/healthcare organization and transplant center (6 items) will include the location (i.e., region), university/non university hospital, type of organ(s) (kidney, liver, heart, lung, other) and patients (adult/pediatric) transplanted, number of transplants performed over the past year, indicative share of immigrant patients transplanted over the past 5 years. The following dimensions will be assessed using the same 5-point Likert scales as for ICU HCPs: (Inter)cultural awareness, sensitivity, knowledge and attitudes (7 items), Practices and behaviors (12 items), Patient-centered communication (14 items), and Usefulness of specific interventions (10 items). Two open-ended qualitative questions will finally serve to acquire additional comments and suggestions to improve the process of transplant education and follow-up management of foreign patients and improve the overall transplant process among this patient population.

Data collection

After providing formal consent to participation in the study and to the processing of personal data, individuals from ethnic and faith communities and ICU and transplant centers' HCPs will be eligible for participation in the study. All data will be collected through a self-administered questionnaire on an online platform entirely managed and safely stored by the CNT.

Ethnic and faith communities

The presentation of the FAITH project and the dissemination of the questionnaire directed towards ethnic and faith communities will be sent by the CNT to regional transplant coordinators, patient and donor associations, and ethnic and faith representatives in the stakeholders working group. Regional transplant coordinators (or their delegates) will partner with local institutional authorities and with local ethnic and faith representatives. A co-design approach will be adopted. Therefore, ethnic and faith representatives will instruct on the most appropriate methods and contexts/types of community-based informative events for project presentation and questionnaire dissemination. Dissemination will be performed also by local patient and donor associations, and other institutional actors operating in the different regions involved in the project, so as to further adapt these initiatives to the specific features of the different local areas. During the informative community-based events, links to the questionnaire - in the different available languages - will be shared with participants via QR codes that will be displayed at the venues where the events will be held. By doing so, participants will have the opportunity, should they wish, to fill out the questionnaire on site, taking the opportunity to ask for more information and/or clarification from the people involved in the project (the project coordinator and/or the General Manager of the CNT - or their delegates - will guarantee their presence during the events). However, participation in the study is voluntary and, therefore, there will be no obligation to complete the questionnaire.

The flowchart of the presentation of the FAITH project and questionnaire dissemination is reported in **Supplementary Figure S2, Panel A**).

ICUs' and Transplant centers' healthcare professionals

As illustrated in **Supplementary Figure S2, Panel B and Panel C**, the CNT will send the project presentation, together with the informative sheets directed towards ICUs' and transplant centers' HCPs to the Welfare General Directorates of the Regions involved in the project and/or to regional transplant coordination centers, which, in turn, will contact the General Directorates of the Hospitals located in their respective Regions. The General Directorates, through local organ procurement and transplant coordinators will disseminate the following documentation to a completely anonymous mailing list (recipients will all be listed in Blind Carbon Copy, BCC) of the HCPs working in the ICUs and transplant centers, of the respective healthcare organizations, respectively: project brochure, informative sheet and link to the questionnaire.

Data analyses

After all data are collected, analysis will be performed using Stata (Stata 17.0, StataCorp LLC Texas TX 77845), R (R 3.6.1). Statistical analyses will be performed separately for the three distinct databases corresponding to the target

populations. Descriptive statistics will be given as total number and percentage for categorical or qualitative variables; median and interquartile range (IQR) otherwise. Associations between categorical variables will be evaluated by chi-square test; Fisher's exact test will be preferred in case of sparse tables. Exploratory data analysis will be performed to examine the relationships between the different variables through clustering and graphical methods (multivariate visualizations) to map the interactions between dimensions. The multivariate models provided for the Structural Equation Modeling scales of the administered questionnaires will also be applied for the evaluation of the objectives, and correlations with the main demographic and identifying group (i.e., ethnic and faith communities, and ICU and transplant center's HCPs) will be assessed with reference to the dimensions in the questionnaires. Factor Analysis models will be adopted (exploratory, and, when this will apply, confirmatory) with the construction of correlation matrices, after the evaluation of some indices (Kaiser-Meyer-Olkin index for adequacy of sampling and Bartlett's Sphericity index) to verify the application of models with Varimax rotation. The models' goodness of fit will be assessed based on Cronbach's alpha.

Expected outcomes

Studies will report on the findings of the project and provide additional insights of the interactions between factors at the individual, interpersonal and societal levels in relation to living will manifestation about organ donation. The collected data will serve to inform the future development of informative initiatives directed toward different ethnic and faith communities in Italy. Further, the data will allow the identification of potential areas for intervention both at the educational level (i.e., development of communicative/relational skills of ICUs' and transplant centers' HCPs) and at the structural/organizational level (*diversity responsiveness* of health care organizations). At the same time, the study results will enable the development of a list of recommendations – which will be shared and further developed and refined with all stakeholders – on how to enable shared decision-making at the different stages of the ODT process for the prevention of inequities for individuals with an immigration background in Italy. The FAITH project will serve also to stimulate the debate surrounding ODT within and among different ethnic and faith communities, to promote and enhance dialogue among all stakeholders, and, ultimately, to increase awareness about the core issues of this study among all of the actors involved.

Research to assess and report on the actual impact of the FAITH project on existing inequities in ODT among immigrant communities in Italy in the longer term are warranted.

Future, qualitative studies including in-depth interviews and/or focus group discussions with bereaved family members, patients pursuing transplant, and (potential) living donors from migrant minority communities will further explore barriers to care.

Ethics and dissemination

The study protocol was approved by the *National ethics committee for clinical trials of public research bodies and other national public institutions* of the Italian National Institutes of Health, Rome, Italy (Protocol number: 0024027 - 19/05/2023).

Three information sheets presenting the overall study objective, and, respectively, the distinct specific objectives for the three target groups will be provided for all participants. Acceptance of the informative sheet and of the data protection form will be obtained from all participants. Further, to improve understanding, the informative sheets and the questionnaire directed towards immigrant communities will be translated in the languages of the more numerous populations in Italy (**Supplementary Table S1**). The collected data will be retained for as long as necessary during the study period, but no longer than 12 months after its completion. After that time, the data will be completely anonymized and retained indefinitely. All collected data and information will be stored on the servers of the CNT according to the security measures related to its management. The data will be analyzed and subsequently published in aggregate form and, in no case, will the information be traced back to individual subjects and, with reference to HCPs, neither to individual subjects nor to individual healthcare organizations.

The study will be conducted in accordance with the ethical principles set forth in the *Nuremberg Code*, the *Declaration of Helsinki*, and the *Belmont Report*, as per recommendations of the European Commission Guide to Ethics in Social Science and Humanities.

The study results will be disseminated through scientific publications, conference communications, publication on the CNT website, and shared during events organized by the CNT and other partners of the FAITH project.

References

1. Health Research & Educational Trust. Becoming a Culturally Competent Health Care Organization [Internet]. 2013. Available from: https://www.aha.org/system/files/hpoe/Reports-HPOE/becoming-culturally-competent-health-care-organization.PDF
2. Grossi AA, Cardillo M. Il Progetto Migrant and ethnic minority Education on Transplantation and Organ donation and process Optimization (ME TOO). Trapianti. 2021 Jul 1;25(3):73–85.
3. Grossi AA, Paredes D, Palaniswami V, Jansen N, Picozzi M, Randhawa G. “One size does not fit all” in organ donation and transplantation: Targeting and tailoring communication for migrant and ethnic minority populations. Commun Med. 2023;18(3):241–57.
4. Morgan SE, Stephenson MT, Harrison TR, Afifi WA, Long SD. Facts versus “Feelings”: how rational is the decision to become an organ donor? J Health Psychol. 2008;13(5):644–58.
5. Morgan SE, Miller JK. Beyond the organ donor card: the effect of knowledge, attitudes, and values on willingness to communicate about organ donation to family members. Health Commun. 2002;14(1):121–34.
6. Khoshravesh S, Karimi-Shahanjarini A, Poorolajal J, Barati M, Bashirian S, Hamidi M, et al. Development and Psychometric Testing of the Signed Donor Card (SDC) Scale in an Islamic Society. Health Commun. 2021;36(8):1029–38.
7. Betancourt JR. Defining Cultural Competence: A Practical Framework for Addressing Racial/Ethnic Disparities in Health and Health Care. Public Health Rep. 2003 Jul 1;118(4):293–302.
8. Schwarz JL, Witte R, Sellers SL, Luzadis RA, Weiner JL, Domingo-Snyder E, et al. Development and psychometric assessment of the healthcare provider cultural competence instrument. Inquiry. 2015;52.
9. Morgan M, Kenten C, Deedat S, Farsides B, Newton T, Randhawa G, et al. Increasing the acceptability and rates of organ donation among minority ethnic groups: a programme of observational and evaluative research on Donation, Transplantation and Ethnicity (DonaTE). Program Grants Appl Res. 2016 Mar;4(4):1–196.
10. Kentish-Barnes N, Siminoff LA, Walker W, Urbanski M, Charpentier J, Thuong M, et al. A narrative review of family members’ experience of organ donation request after brain death in the critical care setting. Intensive Care Med. 2019 Mar 13;45(3):331–42.
11. Skelton SL, Waterman AD, Davis LSA, Peipert JD, Fish AF. Applying best practices to designing patient education for patients with end-stage renal disease pursuing kidney transplant. Prog Transplant. 2015;25(1):77–90.
12. Rubinelli S, Diviani N. The bases of targeting behavior in health promotion and disease prevention. Patient Educ Couns. 2020 Dec 1;103(12):2395–9.
13. International Organization for Migration (IOM). Glossary on Migration [Internet]. 2019. Available from: https://publications.iom.int/system/files/pdf/iml_34_glossary.pdf
14. European Commission. “Migrant” definition [Internet]. Available from: https://home-affairs.ec.europa.eu/pages/glossary/migrant_en
15. Bhopal R. Glossary of terms relating to ethnicity and race: for reflection and debate. J Epidemiol Community Health. 2004;58(6):441–5.
16. Grossi AA, Randhawa G, Jansen NE, Paredes D. Taking a “care pathway/whole systems” approach to Equality Diversity Inclusion (EDI) in organ donation and transplantation in relation to the needs of “ethnic/racial/migrant” minority communities:a statement and a call for action. Transpl Int. 2023;36.
17. EUPHA. Migration, ethnicity and health. Statement drafted by the Section for Migration, Ethnicity and Health of the European Public Health Association (EUPHA). 2018.
18. Schaeffer RL, Mendenhall W, Ott RL. Elementary Survey Sampling. Fourth Edi. Belmont, California: Duxbury Press; 1990.

Supplementary Figure S1. Absolute number of foreign residents by Italian region

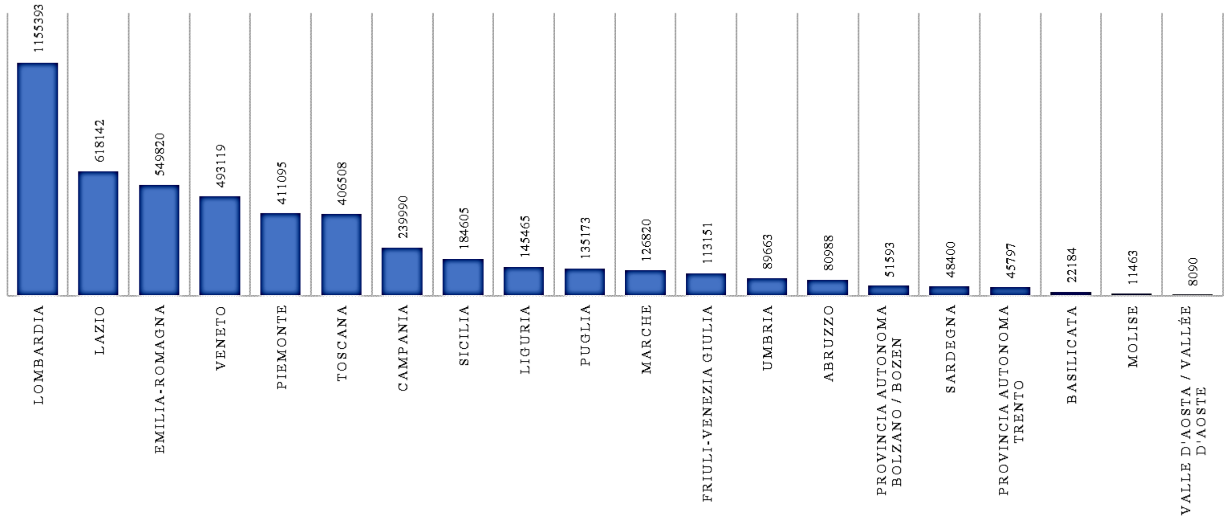

Source: Italian National Institutes of Statistics (ISTAT) (01/01/2022)
http://dati.istat.it/Index.aspx?DataSetCode=DCIS_POPSTRRES1

Supplementary Figure S2. Flowchart of the FAITH project presentation and questionnaires dissemination

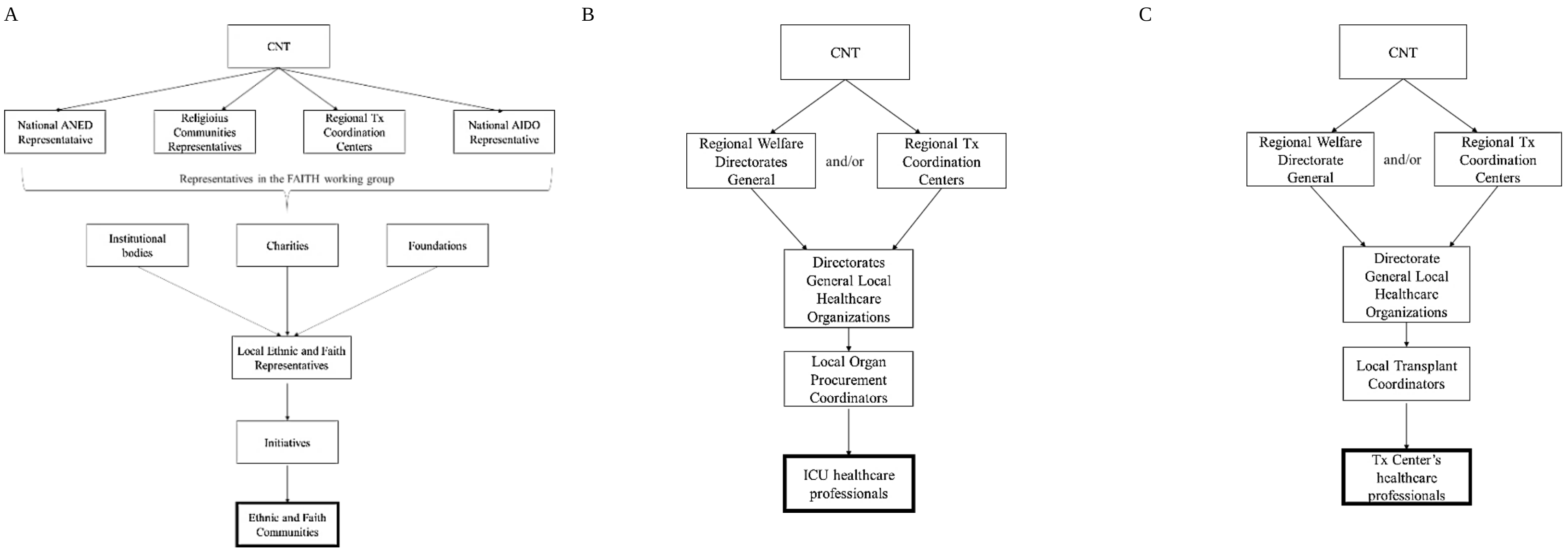

The figure illustrates the flowchart of project presentation and questionnaires dissemination to: ethnic and faith communities (Panel A); ICU healthcare professionals (Panel B); Transplant Centers' healthcare professionals (Panel C).

AIDO, Italian Association for the Donation of organs, tissues and cells; ANED, National Association for Hemodialysis Patients and Transplant Recipients; CNT, Italian National Transplant Center; ICU, Intensive Care Unit; Tx, Transplant

Supplementary Table S1. Distribution of immigrant populations based on citizenship

Country of birth	National representativeness	Representativeness in the regions participating in the project
Romania	1.076.412	859.875
Albania	433.171	324.834
Morocco	428.947	319.690
China	330.495	277.443
Ukraine	235.953	158.325
India	165.512	135.122
Philippines	165.443	148.759
Bangladesh	158.020	123.788
Egypt	139.569	132.989
Pakistan	135.520	102.738
Moldova	122.667	108.457

The table reports only the most represented populations

Source: Italian National Institutes of Statistics (ISTAT) (01.01.2021)

Supplementary Table S2. Stakeholders' feedback on barriers to communication, education and management of immigrant and ethnic minority populations and individuals in the ODT process

	Individual Level			Interpersonal Level	Societal Level	
	Cognitive	*Personal*	*Psychological*		*Healthcare Institutions*	*Social context and policies*
**P1**	• Lack of knowledge of:<br>- modality of living will expression<br>- deceased donation<br>- certainty of death at time of organ procurement<br>- position of one's religion toward organ donation<br>- susceptibility to Tx<br>- ability to receive Tx<br>- ODT process<br>- legislative regulations surrounding ODT<br>- ODT system<br>- host country language<br><br>• Inferior:<br>- educational level<br>- health literacy<br>- language proficiency	• Knowledge of:<br>- individuals who have previously received Tx<br>- individuals who have pursued LD<br><br>• Prior experiences:<br>- negative experiences in healthcare<br>- organ trafficking (especially applied to forced migrants)<br>- body violence (especially applied to forced migrants)	• Lack of trust toward:<br>- HCPs<br>- healthcare institutions<br>- healthcare system<br><br>• Fear of:<br>- violation of body integrity<br>- discrimination<br>- body violence and subsequent representation of the body (forced migrants)<br>- organ trafficking (forced migrants)<br>- speaking about death	• Different representation of:<br>- death<br>- ethical aspects<br>- symbolic value of donation<br>- good clinical practice/care<br><br>• Reluctance to speak about death within family units	• Lack of:<br>- culturally and linguistically appropriate informative material<br>- migrant-friendly and culturally competent healthcare services<br>- culturally competent end-of-life care<br>- targeted initiatives for the early management of chronic conditions	• Discrimination<br>• Marginalization<br>• Inequities<br>• Poor integration and integration policies<br>• Precarious housing/working conditions<br>• Lack of recognition of religious identity
**P2**	• Lack of knowledge of:<br>- deceased donation<br>- certainty of death<br>- susceptibility to Tx<br>- ability to receive Tx<br>- Tx process<br>- legislative regulations surrounding ODT<br>- ODT system<br>- host country language<br><br>• Inferior:<br>- educational level<br>- health literacy<br>- language proficiency	• Knowledge of:<br>- individuals who have previously received Tx<br>- individuals who have pursued LD<br><br>• Prior experiences:<br>- negative experiences in healthcare<br>- organ trafficking (especially applied to forced migrants)<br>- body violence (especially applied to forced migrants)	• Lack of trust toward:<br>- HCPs<br>- healthcare institutions<br>- healthcare system<br><br>• Fear of:<br>- death (inability to accept it)<br>- violation of body integrity<br>- discrimination<br>- disattending the wishes of the deceased family member<br>- disattending religious/spiritual positions regarding organ donation<br>- not having the opportunity to see one's beloved person after donation<br>- body violence and subsequent representation of the body (forced migrants)<br>- organ trafficking (forced migrants)	• Different representation of:<br>- death (brain/cardiac death)<br>- body (i.e. body integrity)<br>- dying process<br>- "good" end-of-life care<br>- consent (i.e. family-mediated)<br>- decision-maker (i.e. legal vs. cultural decision-maker)<br><br>• Difficulties for HCPs:<br>- management of large family units<br>- difficult identification of reference person<br>- difficult identification of family decision-maker<br>- difficult achievement of shared decision by family members	• Healthcare professionals:<br>- lack of HCPs' ODT training<br>- lack of HCPs' diversity-sensitive/culturally competent communication training<br>- lack of HCPs' time due to busy hospital schedules<br><br>• Cultural mediators:<br>- lack of specific training on ODT<br>- difficulty of involving cultural mediators given unstable working conditions (i.e. they are most frequently employed in external organizations and involving them often takes too much time)<br><br>• ICU/local transplant coordination center:<br>- lack of multilingual informative material<br>- lack of professional mediation and/or translation services	

					- lack of contacts with local religious/spiritual community referents<br>- fragmented communication with multiple physicians in ICUs based on hospital shifts making it difficult to build trust<br>- lack of adequate communication settings for organ donation conversations<br>- lack of “multicultural/multi-faith” spaces for family gatherings	
**P3**	• Lack of knowledge of:<br>- Tx process<br>- risks and benefits of Tx<br>- risks and benefits of LD<br>- legislative regulations surrounding ODT<br>- organizational ODT system<br>- NHS accessibility and functioning<br><br>Inferior:<br>• Educational level<br>• Health literacy<br>• Language proficiency	• Knowledge of<br>- individuals who have previously received Tx<br>- individuals who have pursued LD	• Fear of:<br>- Tx<br>- accepting a “foreign” organ within oneself	• Different representation of:<br>- Tx (i.e. complete remission from illness)<br>- medications (with the potential to affect adherence to post-Tx requirements)<br>- healthy lifestyle, including dietary habits	• Healthcare professionals:<br>- fragmented communication with multidisciplinary HCPs making it difficult to build trust<br>- lack of training on culturally competent communication in ODT<br>- lack of diversity sensitivity training in ODT<br>- lack of time to invest into the patient-physician relationship due to busy hospital schedules<br><br>• Cultural mediators:<br>- lack of specific training on ODT<br>- difficulty of involving cultural mediators given unstable working conditions (i.e. they are most frequently employed in external organizations and involving them often takes too much time)<br><br>• Transplant centers:<br>- lack of multilingual informative material<br>- lack of professional mediation and/or translation services<br>- lack of dedicated desks to facilitate the bureaucratic process and deliver	• Discrimination<br>• Marginalization<br>• Inequities<br>• Poor integration<br>• Precarious housing/working conditions<br>• Inferior social support (i.e. more frequent absence of family members)<br>• Difficult navigation of a complex healthcare system<br>• Lack of diversity-sensitive and culturally competent healthcare services<br>• Bureaucratic/administrative difficulties encountered by potential non-resident LDs

		multilingual informative materials

The table illustrates the feedback received by the stakeholders in the working group of the FAITH project of the CNT answering the following key questions: (1) What are the potential barriers to living will expression? (2) What are the potential obstacles/difficulties most frequently encountered within the relationship between ICU healthcare professionals and bereaved families in the context of organ donation as part of end of life care? (3) What are the potential needs of patients and potential living donors (i.e. gaps in knowledge/understanding of aspects relevant to transplant accessibility and outcome, health beliefs, preferred educational formats, other needs and/or problems encountered)? P1 refers to living will manifestations, P2 to the relationship between ICU healthcare professionals and potential deceased donor families, and P3 to the relationship between transplant professionals and transplant candidates, recipients and (when this is applicable) their potential living donors. The results have been categorized based on the socio-ecological model (47). We acknowledge that the factors intervening at the different levels have the potential to reciprocally influence one another. However, investigation of these aspects was beyond the scope of this study at this stage of the project.
The stakeholders' working group includes the following representatives: regional and local transplant coordinators, intensive care specialists, transplant center nurses, psychologists, social workers, patient and donor associations, gender-specific medicine experts, foreign-born physicians, transplant recipients with an immigration background, communication and medical ethics experts, education specialists, migration medicine and migrants/refugees' assistance experts, and religious communities The multistakeholder groups were composed respectively by the following stakeholder representatives: (a) cultural mediators, ICU specialists and transplant coordinators, religious communities, patients' and donors' associations, communication specialists, and bioethics/clinical ethics experts; (b) cultural mediators, ICU specialists, regional and European transplant coordinators, and religious communities; (c) psychologists, social workers, nephrologists, migration medicine experts, and transplant center clinicians and nurses. These data were preliminarily presented as an oral presentation at the 45th conference of the Italian Society of Organ Transplantation (SITO) in Trieste, Italy (October 23-25, 2022).

CNT, Italian National Transplant Center; HCP, Healthcare Professionals; ICU, Intensive Care Unit; LD, Living Donation; NHS, National Healthcare System; ODT, Organ Donation and Transplantation; P, Process; Tx, Transplantation

Supplementary Table S3. Stakeholders' feedback on potential interventions to improve communication, education and management of migrant and ethnic minority populations/individuals in the ODT process

P1 (Institutional actors ↔ public/communities)<br>Improving communication about ODT among the public	*P2 (ICU HCPs ↔ bereaved family members)*<br>Improving relational processes in ICUs	*P3 (Tx center's HCPs ↔ patients/potential LDs)*<br>Improving patient education and management at Tx centers
• Public communication and information:<br>- drawing on testimonials from different ethnic and faith groups to tell their own experiences of ODT (either in-person, during community-based educational interventions or video-based)<br>- defining more appropriate information and dissemination methods through discussion with civil ethnic and faith leaders<br>- improving information about ODT among the children (either native- or foreign-born) of foreign-born families (promote school initiatives)<br>- actively engage ethnic and faith community leaders/migrant and ethnic minority individuals who have received Tx to be ODT ambassadors within their communities<br>- translating ODT informative materials into different languages<br>- sharing historical paths on the topic of ODT among different faith communities<br>- fostering and improving dialogue and trust among all stakeholders in ODT<br>- improving health literacy among migrant and ethnic minority communities (i.e. by inclusion of healthcare education in Italian as a second language schools)	• Education and training:<br>- training and education of ICU HCPs for the provision of culturally competent discussions about organ donation as part of end-of-life care<br>- training and education of HCPs on specific cultural aspects of different ethnic and faith communities<br>- training and education of cultural mediators on ODT<br><br>• Informative materials:<br>- development of informative materials for ICUs (printed resources, videos in different languages, information brochures in waiting rooms) by participation and inclusion of ethnic and faith representatives<br><br>• Relational improvements for HCPs:<br>- work in synergy with the cultural mediator (i.e. discuss with cultural mediator before interviewing family members)<br>- use simple and clear language<br>- use visual aids to enhance understanding of brain death (i.e. x-ray images, simple drawings, or other visual material)<br>- use multilingual informative material<br>- pay attention to non-verbal cues (i.e. voice tone, face expressions<br>- verify understanding (i.e. ask family members to repeat back the contents of communication using their own words)<br>- establish trusting relationship with family members by showing care (ideal to identify a reference person all throughout the process)<br>- make sure that family members are aware of the entire diagnostic and therapeutic process which preceded the determination of death of their beloved one<br>- inform about the procedures for the management of the deceased person's body after the determination of death<br>- agree and be open to speak with all of the family members that the family asks to be present during organ donation conversations<br>- identify the decision-maker within the family unit (both the legal and, if this applies, also the cultural one)<br>- when this may be needed, perform conversations with both female and male HCPs<br>- reassure family members that the body of their beloved one will be treated with care and respect in the event of donation	• Trust building:<br>- improve prevention and/or early care of chronic conditions to increase confidence towards the healthcare system and HCPs<br><br>• Transplant education:<br>- share multilingual informative material about treatment options for ESKD: hemodialysis, peritoneal dialysis, deceased donor and living donor kidney Tx early in the process (i.e. at nephrology and dialysis outpatient clinics)<br>- use simplified informative material (i.e. bullet-point lists, comics)<br>- deliver information gradually and verify understanding (i.e. ask the patient and family members to repeat back the contents of information using their own words) at each encounter<br>- ask the patient about his/her favorite educational format (i.e. in-person conversations, paper- or video-based educational material for use outside of clinical encounters)<br>- elicit the patient's and patient's family's representation of organ donation (living and deceased) and Tx<br>- when this may be needed, perform conversations with both female and male HCPs<br>- create personalized objectives according to the patient's individual circumstances<br>- develop pathways to transplant information in group settings, also involving other couples who have already gone through the LD process<br>- develop and expose patients and families to video-based testimonials (i.e. from patients who have already received Tx about improvements in quality of life after Tx and the need for adherence to complex post-Tx regimens)<br>- develop counseling and educational strategies adapted to the specific needs of migrant and ethnic minority populations and further tailor them to the individual needs (i.e. according to level of health literacy, education, language proficiency, individual life circumstances, etc.)<br>- create a network of shared experiences to improve education and provide ongoing social and psychological support<br><br>• Psychosocial assessments:<br>- identify logistical, structural and socioeconomic problems early in the process so as to enable planning of appropriate management of pre- and post-Tx requirements<br>- explore the individual migration projects so as to implement strategies to enable optimal follow-up in the event of willingness to emigrate to another country

		- practice active listening with bereaved family members to elicit their individual views and needs about end-of-life care<br>- offer the opportunity to involve a representative of the reference religious/spiritual community or of the healthcare organization<br>- respect and strive to meet religious/spiritual care requests<br>- choose an appropriate communicative setting for the donation conversation (choose a neutral and appropriate place)	- explore the patient's and family's knowledge of available treatment options and their beliefs about them<br>- elicit the patient's and family's representation and beliefs about medications, lifestyle factors and dietary habits with the potential to compromise adherence to pre- and post-Tx regimens<br>- establish support groups at Tx centers<br>- improve information about Tx and ensure good follow-up management<br>- identify trusted support person to accompany the Tx process<br>- inform, educate and involve the patient's family in the process<br><br>• Bureaucratic procedures:<br>- create dedicated desks to facilitate the bureaucratic process and deliver multilingual informative materials

The table illustrates the feedback received by the stakeholders in the working group of the FAITH project of the CNT answering the key question of how to potentially intervene to enhance communication, education and management of migrant and ethnic minority populations/individuals at the three different stages of the ODT process. These initiatives require the participation and inclusion of migrant and ethnic minority communities and individuals (i.e. patients and, when this applies, potential living donors) at all stages of the process (from the development through to the evaluation stage).

CNT, Italian National Transplant Center; ESKD: End Stage Kidney Disease; HCP, Healthcare Professionals; ICU, Intensive Care Unit; LD, Living Donation; NHS, National Healthcare System; ODT, Organ Donation and Transplantation; P, Process; Tx, Transplantation
